# In situ click chemistry generation of cyclooxygenase-2 inhibitors

**DOI:** 10.1038/s41467-016-0009-6

**Published:** 2017-02-23

**Authors:** Atul Bhardwaj, Jatinder Kaur, Melinda Wuest, Frank Wuest

**Affiliations:** 1 Department of Oncology, University of Alberta, 11560 University Avenue, Edmonton, Alberta Canada T6G 1Z2; 2 Faculty of Pharmacy and Pharmaceutical Sciences University of Alberta, 8613-114 Street, Edmonton, Alberta Canada T6G 2H7

## Abstract

Cyclooxygenase-2 isozyme is a promising anti-inflammatory drug target, and overexpression of this enzyme is also associated with several cancers and neurodegenerative diseases. The amino-acid sequence and structural similarity between inducible cyclooxygenase-2 and housekeeping cyclooxygenase-1 isoforms present a significant challenge to design selective cyclooxygenase-2 inhibitors. Herein, we describe the use of the cyclooxygenase-2 active site as a reaction vessel for the in situ generation of its own highly specific inhibitors. Multi-component competitive-binding studies confirmed that the cyclooxygenase-2 isozyme can judiciously select most appropriate chemical building blocks from a pool of chemicals to build its own highly potent inhibitor. Herein, with the use of kinetic target-guided synthesis, also termed as in situ click chemistry, we describe the discovery of two highly potent and selective cyclooxygenase-2 isozyme inhibitors. The in vivo anti-inflammatory activity of these two novel small molecules is significantly higher than that of widely used selective cyclooxygenase-2 inhibitors.

## Abstract (web summary)

Traditional inflammation and pain relief drugs target both cyclooxygenase 1 and 2 (COX-1 and COX-2), causing severe side effects. Here, the authors use in situ click chemistry to develop COX-2 specific inhibitors with high in vivo anti-inflammatory activity.

## Introduction

Cyclooxygenase (COX) enzymes catalyze the metabolic conversion of arachidonic acid to prostanoids including prostaglandins (PGs), prostacyclin, and thromboxane, which play important roles in human physiology and various pathological conditions^1–4^. Despite several known side effects like myocardial infarction and atherothrombotic events, drugs aimed at COXs inhibition is a billion dollar industry, inspiring scientists to search constantly for novel COX inhibitors. COX exists in three isoforms: cyclooxygenase-1, 2, and 3 (COX-1, COX-2, and COX-3)^5–7^. COX-1 and COX-2 isoforms are of primary interest, as they are involved in physiological as well as pathological processes. COX-1 is a constitutively expressed house-keeping isozyme responsible for the basal production of essential PGs^8^. These PGs mediates homoeostatic functions in the gastrointestinal and cardiovascular system. COX-3 (a splice variant of COX-1) is expressed only in specific parts of the brain and spinal cord and its exact functions are still unclear^9^. In contrast, COX-2 isozyme is expressed at very low levels under normal conditions. However, COX-2 expression is rapidly upregulated in the immediate response to diverse pro-inflammatory and pathogenic stimuli. There is accumulating evidence for the critical involvement of COX-2 in various pathologies that include inflammation^10,11^, cancer^12–14^, neurodegenerative diseases^15^ and multidrug resistance^16^. Therefore, beyond their traditional use as anti-inflammatory agents, COX-2 inhibitors have recently been used for molecular imaging^17–19^ and therapy^20–22^ of cancer. Hence, the development of selective COX-2 inhibitors as anti-inflammatory and anti-tumor drugs is a major direction in academic research and pharmaceutical industry^23–25^. Traditional nonsteroidal anti-inflammatory drugs (NSAIDs) (aspirin, ibuprofen, naproxen) inhibit both COX-1 and COX-2 isoforms; and their use is limited due to associated ulcerogenic and gastrointestinal side effects. Discovered in the late 1990’s, COX-2 selective inhibitors (the COXIBs: celecoxib, rofecoxib) are diarylheterocycles possessing a SO_2_NH_2_ or SO_2_Me group as COX-2 pharmacophore, which exert similar anti-inflammatory and antipyretic properties as traditional NSAIDs but are devoid of gastrointestinal toxicity^4^. However, COXIBs are also under scrutiny since several studies have demonstrated that chronic use of COXIBs can elevate the risk of myocardial infarction and other thrombotic events by stalling the biosynthesis of anti-aggregatory prostacyclin (PGI_2_) while leaving the biosynthesis of pro-thrombotic thromboxane A_2_ (TxA_2_) unaffected^26–29^. As a result, COXIBs such as rofecoxib and valdecoxib were withdrawn from the market thereby leaving a demand for the synthesis and screening of novel COX-2 inhibitors.

Development of compounds that selectively inhibit COX-2 over COX-1 is a substantial challenge as both isoforms share similar cellular expression locations, molecular weight, and amino-acid composition. In addition, the both isoforms share more than 60% sequence homology and their three-dimensional structures are almost superimposable. However, the key difference between the COX-1 and COX-2 isozyme active site is the exchange of isoleucine in COX-1 for valine in COX-2 at positions 434 and 523. The difference in the amino-acid sequence make the COX-2 substrate-binding site more flexible and approximately 25% larger by creating a distinct secondary-binding pocket^3,30^. Many COX-2 selective inhibitors explicitly bind to this secondary-binding pocket (lined by H90, R513, and V523) resulting in the specific inhibition of COX-2 activity. Another important region in the COX-2 active site is the hydrophobic pocket (lined by W387, Y385, F518, F381, L352), and a recent mutational study described the involvement of hydrophobic pocket residues in the proper positioning of fatty acid substrates for oxygenation^31^. Therefore, highly potent and selective COX-2 inhibitors should possess a pharmacophore which can selectively bind in the secondary pocket and deliver sufficient steric bulk to block the hydrophobic channel of COX-2.

Here, we deviated from conventional drug discovery approaches involving the laborious synthesis and screening of a range of compounds, and envisioned to explore the utility of in situ click chemistry for the discovery of specific and high-affinity COX-2 inhibitors. Click chemistry^32,33^, including 1,3-dipolar cycloaddition between alkyne and azide (Huisgen cycloaddition), have attracted much attention because of their remarkable efficiency, simplicity, and its ability to be employed for the synthesis of a wide range of compounds such as molecular imaging agents^34,35^ and drugs^36^, protein modification^37,38^, DNA and RNA targeting^39,40^, and glycan imaging^41,42^. Considering its versatility, click chemistry has found numerous synthesis applications not only performed in traditional reaction vessels, but also in living systems. Kinetic target-guided synthesis (TGS), also termed as in situ click chemistry is an innovative synthesis process where a biological target assembles its own inhibitor through target-guided selection of appropriate building blocks^43,44^. TGS was elegantly used for the preparation of high affinity inhibitors of enzymes like acetylcholine esterase^45–47^, HIV protease^48^, bovine carbonic anhydrase II^49,50^, protein tyrosine phosphatases^51^, metalloproteases^52,53^, nicotinic acetylcholine receptors^54^, and chitinase^55^.

Herein, we demonstrate the use of the COX-2-binding site as a reaction vessel for generating its own highly potent and selective inhibitors.We designed and synthesized a range of pyrazole-based azide building blocks (**5**, **14**, **27,** and **31**) and a collection of corresponding triazole-containing biheterocyclic compounds (**7**–**12, 16–25, 28, 29, 32,** and **33**). After screening for their COX-1/COX-2 inhibitory potency, various 5-azido-pyrazoles (**5, 14, 27,** and **31**) and aryl acetylenes as click chemistry building blocks were incubated in pairs with human recombinant COX-2 isozyme to test the capability of COX-2 for assembling its own highly potent inhibitors. Identification of compounds **18** and **21** as highly potent and selective COX-2 inhibitors demonstrated the feasibility of the in situ click chemistry approach. Compounds **18** and **21** displayed a superior in vivo anti-inflammatory activity profile compare to clinically used anti-inflammatory drugs. Multi-component competitive-binding studies confirmed that COX-2 can selectively group most appropriate building blocks from a pool of compounds to construct highly potent COX-2 inhibitors. The thermodynamic-binding signatures calculated from isothermal titration calorimetry (ITC) confirmed that binding of click chemistry building block 5-azido-pyrazole (**14**) to COX-2 involves a favorable change in free energy (ΔG = −36.20 kJ mol^−1^), which was mainly based on H-bonding and van der Waals interactions. Moreover, comprehensive computational analysis including structure activity relationship (SAR) and molecular docking indicated that the size and type of the COX-2 pharmacophore, and the orientation of the clickable building blocks inside the binding site of the target protein collectively contribute to the in situ construction of highly potent and selective COX-2 inhibitor.

## Results

### Design of clickable building blocks

**Fig. 1:**
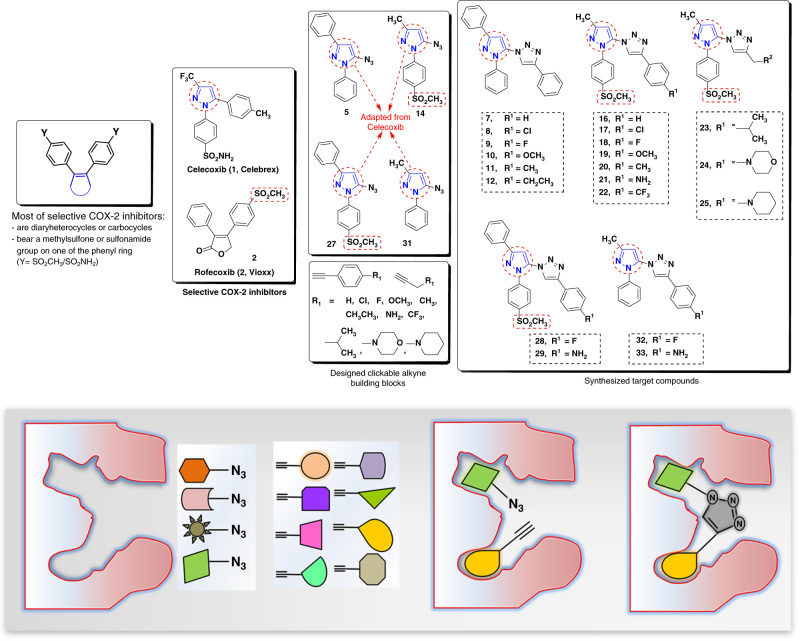
**Design of building blocks for in situ click chemistry reaction in the COX-2 isozyme.** Structure of selective COX-2 inhibitor celecoxib (**1**) and rofecoxib (**2**), target compounds (**7–12**, **16–25**, **28**, **29**, **32,** and **33**) and illustration of in situ click chemistry reaction principle inside the COX-2-binding pocket

Based on the structural features of the COX-2 active site, we concluded that suitable clickable building blocks should meet two criteria: (i) at least one of the building blocks (in our case, the azide component) should possess a SO_2_Me COX-2 pharmacophore to facilitate its tight binding into the secondary-binding pocket of the COX-2 isozyme; (ii) the azide component should have a proper size and orientation that will not interfere with the entry of the alkyne component into the COX-2 active site, allowing the in situ click chemistry formation of a potent and selective inhibitor large enough to block the hydrophobic channel of the COX-2 isozyme (Fig. 1).

Hence, to optimize these parameters we synthesized a range of 5-azido-pyrazole derivatives with variable sizes containing a COX-2 pharmacophore (SO_2_CH_3_) and evaluated the influence of the chemical structure on the success of in situ click chemistry reaction. We selected a pyrazole motif as the central scaffold for the azide building blocks, as pyrazole-containing compounds are quite prevalent in many anti-inflammatory drugs. The design of click chemistry building blocks and target compounds is illustrated in Fig. 1, where the central pyrazole motif was adapted from approved drug celecoxib (**1**). The SO_2_CH_3_ COX-2 pharmacophore is located at the *para* position of one of the phenyl ring which was introduced to facilitate binding of 5-azido-pyrazole (**14**) into the COX-2 secondary-binding pocket. Extensive SAR data is available in the literature, which describes the importance of SO_2_CH_3_ and SO_2_NH_2_ groups as COX-2 pharmacophores for selective binding to the COX-2 isozyme. However, SO_2_NH_2_ groups are also found in many drugs inhibiting members of the carbonic anhydrase family^56^. Substitution pattern on the alkyne building blocks selected for this study was based on structures frequently present in various NSAIDs.

### Chemical synthesis of target compounds

**Fig. 2:**
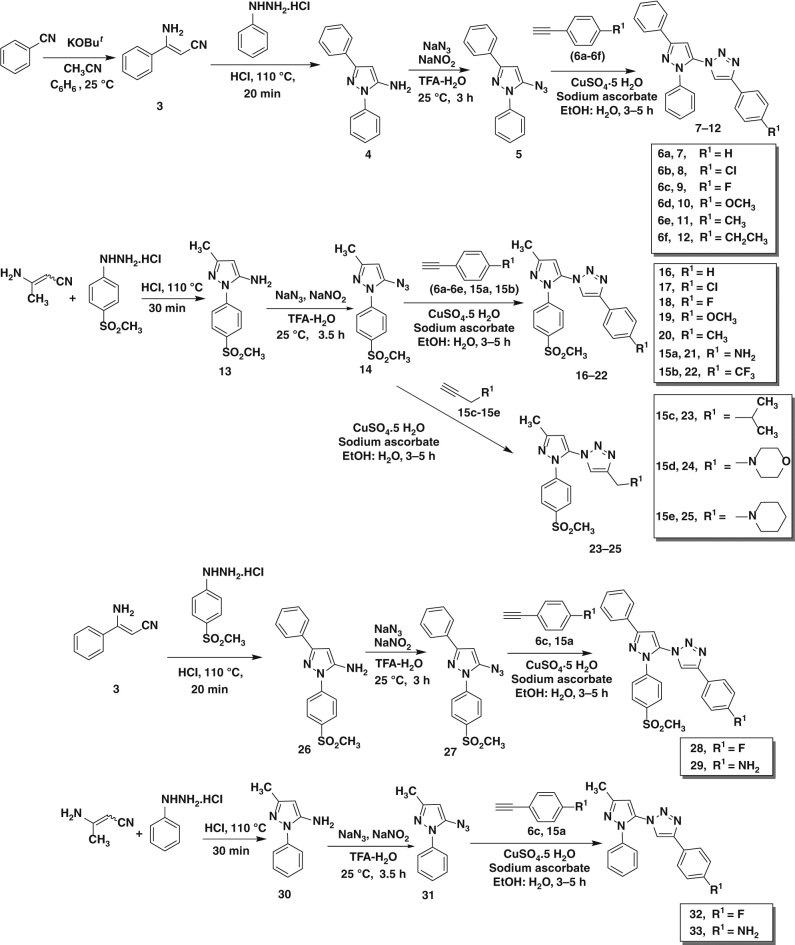
**Synthesis scheme of target compounds.** Synthesis of various 5-azido-pyrazoles (**5**, **14**, **27,** and **31**) and reference compounds (**7–12**, **16–25, 28**, **29**, **32,** and **33**)

The synthetic methodologies used to prepare 5-azido-pyrazoles (**5**, **14**, **27**, **31**) are illustrated in Fig. 2. Briefly, precursor compounds 2,5-diphenyl-2*H*-pyrazol-3-ylamine (**4**) and 2-(4-methane-sulfonyl-phenyl)-5-methyl-2*H*-pyrazol-3-ylamine (**13**) were synthesized in high yields according to known procedures involving the reaction of 3-amino-3-phenyl-acrylonitrile (**3**) or 3-amino-but-2-ene-nitrile with the appropriate arylhydrazine. Subsequently, compounds **4** and **13** were converted into corresponding 5-azido-pyrazoles (**5**, **14**) by diazotization and subsequent treatment with sodium azide. 5-azido-pyrazoles (**5**, **14**) were reacted with various alkynes (**6a**–**6f, 15a**–**15e**) using standard Cu(I)-catalyzed azide-alkyne cycloaddition (CuAAC) reaction conditions. Respective triazole products (**7–12**, **16–25**) were isolated in high yields (Fig. 2). To illustrate the role of SO_2_CH_3_ COX-2 pharmacophore in selective COX-2 inhibition, we synthesized compounds **28**, **29**, **32,** and **33** according to synthetic methods shown in Fig. 2.

In order to investigate the COX-2 isozyme-mediated in situ click chemistry reaction, 5-azido-pyrazole building blocks (**5, 14, 27,** and **31,** 1 µl of 3 mM dimethyl sulfoxide (DMSO) solution) and alkynes (**6a**–**6f, 15a**–**15e,** 1 µl of 20 mM DMSO solution) were incubated as pairs in the presence of the human recombinant COX-2 isozyme (95 µl COX-2) in 1 µl of 1M Tris-HCl, pH 8.0 for 24 h at room temperature. After 3, 6, 9, 12, 15, 18, 21, and 24 h each sample was analyzed in triplicate by injecting (10 µl) into the liquid chromatography–mass spectrometry (LC/MS) instrument with selected-ion-monitoring (SIM) mode. In library 1, where 5-azido-1,3-diphenyl-1*H*-pyrazole (**5**) was incubated in the presence with various alkynes (**6a**–**6f**, **15a**, and **15b** Table 1, library 1), in situ click chemistry formation of corresponding triazole compounds could not be detected. After refinement of the structure of 5-azido-1,3-diphenyl-1*H*-pyrazole (**5**,V_molecular_ = 332.9 Å^3^) into a smaller sized 5-azido-1-(4-methanesulfonyl-phenyl)-3-methyl-1*H*-pyrazole (**14**,V_molecular_ = 326.1 Å^3^), where one of the phenyl ring present at C-5 position of the pyrazole ring was replaced with a CH_3_ group. In addition, a COX-2 pharmacophore (SO_2_CH_3_) was incorporated at C-4 position of one of the phenyl rings.

**Table 1:** In situ click chemistry building blocks and corresponding hit compounds

	

**Fig. 3:**
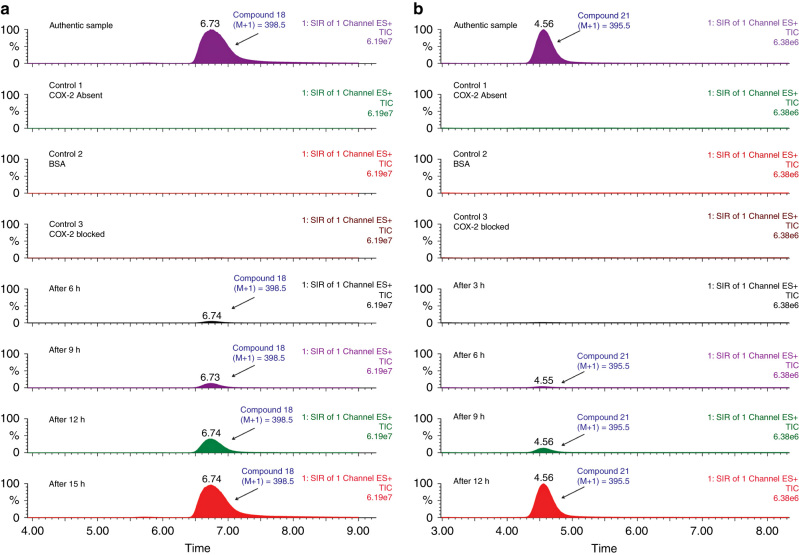
**Monitoring of COX-2 isozyme-mediated in situ click chemistry reaction.** In situ click chemistry formation of compounds **18** (**a**) and **21** (**b**) as determined by LC/MS-SIM

LC/MS-SIM analysis of 11 different reactions (**6a**–**6f, 15a**–**15e** Table 1, library 2) revealed two combinations where in situ click chemistry formation of two compounds, 4-(4-fluorophenyl)-1-[2-(4-methanesulfonyl-phenyl)-5-methyl-2*H*-pyrazol-3-yl]-1H [1,2,3]triazole (**18**) and 4-{1-[2-(4-methanesulfonyl-phenyl)-5-methyl-2*H*-pyrazol-3-yl]-1H-[1,2,3]triazol-4-yl}-phenylamine (**21**) was detected (Fig. 3). Formation of compounds **18** and **21** was first detected at a time interval of 6 h, and continuous elevation in the LC/MS response signal (an indicator for the amount of triazole product formed) was noticed up to 15 and 12 h, respectively.

Likewise, three control experiments were performed under the same experimental conditions to test false-positive results: each azide/ alkyne combination was incubated either in the (a) absence of COX-2 isozyme(b) presence of bovine serum albumin (BSA, 1 mg ml^−1^) as model protein instead of COX-2 isozyme(c) or in the presence COX-2 isozyme and known COX-2 selective inhibitor celecoxib (1 µl of celecoxib, 100 µM final concentration). LC/MS-SIM analysis of each reaction mixture revealed that none of the reagent combinations led to the formation of triazole products. These results demonstrated that desired triazole product **18** and **21** were only formed when a suitable 5-azido-pyrazole and alkyne combination can undergo in situ click chemistry reaction in the presence of an accessible COX-2-binding pocket. In line with other known in situ click chemistry examples^42,43^, our results confirm that the COX-2-binding site can also serve as a reaction vessel for 1,3-dipolar cycloaddition reactions, and this innovative methodology can facilitate the discovery and quick screening of novel selective COX-2 inhibitors.

The 1,4/1,5 triazole regiochemistry of triazole products (**18** and **21**) formed during the COX-2 mediated in situ click chemistry reaction was determined through LC/MS analysis comparing the retention time of 1,4-triazole regioisomers (compound **18** and **21**) with respective 1,4/1,5 triazole regioisomer mixtures of compounds **18** and **21** (Supplementary Figs 1 and 2). Results from this study demonstrated that in situ click chemistry reaction in the presence of the COX-2-binding site was highly regioselective, yielding the desired 1,4-triazole regioisomers exclusively.

**Fig. 4:**
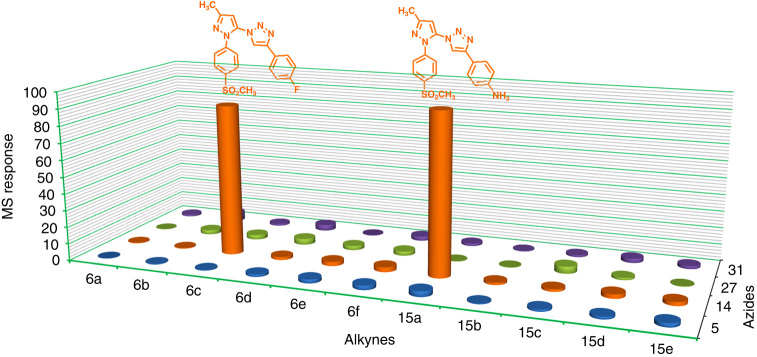
**Multicomponent in situ click chemistry reaction.** Comparison of triazole compounds formed after the incubation of all azide and alkyne building blocks in the presence of the COX-2 isozyme

Furthermore, the influence of the size and the role of the SO_2_CH_3_ COX-2 pharmacophore of the 5-azido-pyrazole building blocks upon in situ click chemistry reaction was studied with two newly synthesized 5-azido-pyrazole building blocks **27** and **31** (Fig. 2). In contrast to 5-azido-pyrazole (**14**), the new building block **31** lacks the SO_2_CH_3_ COX-2 pharmacophore. Upon incubating 5-azido-pyrazole (**31**) with alkynes (**6c** or **15a**) in the presence of COX-2 isozyme, no triazole product formation was detected even after 36 h. This experiment demonstrated that (for library 3) the presence of the SO_2_CH_3_ COX-2 pharmacophore is an important structural requirement for the appropriate positioning of 5-azido-pyrazole building block (**14**) inside the COX-2-binding site to achieve a suitable steric orientation of the azide building block needed for the COX-2 mediated in situ click chemistry reaction. Another set of experiments involved the incubation of phenyl group-containing 5-azido-pyrazoles **27** (with SO_2_CH_3_ COX-2 pharmacophore) and **5** (without SO_2_CH_3_COX-2 pharmacophore) as examples for larger size azide building blocks with the alkyne building blocks (**6c** or **15a**) in the presence of COX-2 isozyme. And in none of the reagent combinations, no in situ generation of expected triazole product was detected. These results led to the following important two conclusions: (*i*) the presence of the SO_2_CH_3_ COX-2 pharmacophore is the defining factor to advance the in situ click chemistry reaction between azides and alkynes inside the COX-2-binding site; (*ii*) a larger sized (additional steric bulk)5-azido-pyrazole building block like compound (**27**) greatly reduces the possibility to achieve an in situ click chemistry reaction in the COX-2-binding site. The results of in situ click chemistry reaction with various azides (**5, 14, 27,** and **31**) and alkynes (**6a-f**, **15a-e**) in the presence of the COX-2 isozyme are summarized in Fig. 4.

We also tested whether COX-2 isozyme is capable of selecting the most suitable compounds from a combined mixture of different azides and alkynes building blocks for assembling the “best-fit inhibitor.” For this purpose, each azide (**5, 14, 27,** and **31,** 1 µl of 3 mM DMSO solution) was mixed with eleven alkynes (**6a–6f** and **15a–15e,** 1 µl of 20 mM DMSO solution) in the presence of COX-2 isozyme (95 µl COX-2) in 1 µl of 1 M Tris-HCl, pH 8.0 and incubated for 24 h at room temperature. After incubation for 24 h, only expected triazole products (**18**, **21**) were detected, demonstrating that COX-2 isozyme can sense small structural differences of the building blocks enabling selection of suitable building blocks for the in situ click chemistry formation of high affinity and selective COX-2 inhibitors (Fig. 4). The amount of triazole product **21** formed seemed to be higher compared to triazole **18** (estimated from the mass response), although the yield of both triazole products (**18**, **21**) was lower (almost 50% reduced) compared to previously performed in situ click chemistry reaction when both building blocked were used as reaction pairs. The decrease in yield could be attributed to a reduction in the concentration of click chemistry building blocks and/or an increase in the competition for the COX-2-binding site.

In parallel to the COX-2 isozyme mediated in situ click chemistry reactions, similar experiments were also performed using COX-1 isozyme. However, no in situ click chemistry reaction to form the desired triazole products was not observed in any of the used reagent combinations. As demonstrated in a later in vitro COX inhibitory-binding assay, none of the compounds displayed inhibitory potencies in the studied concentration range towards COX-1 isozyme. Thus, in situ click chemistry can also be used for the identification of highly selective COX-2 inhibitors. Compound **5**, **14, 27,** and **31** were also studied for their stability in human and rat serum at 37 °C, and no significant degradation was observed up to 30 h (see Supplementary Fig. 3). The stability of hit compounds (**18** and **21**) was tested, and the percentage of intact compound at various time points is given in Supplementary Fig. 3.

### Isothermal titration calorimetry

**Fig. 5:**
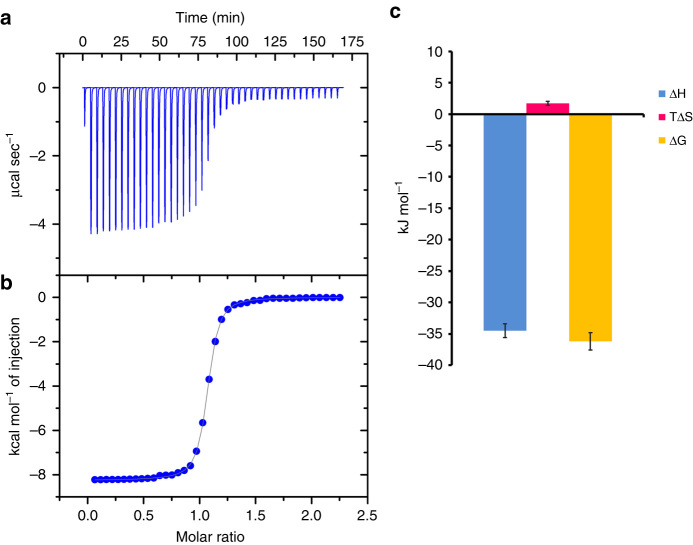
**Thermodynamic analyses for the binding of 5-azido-pyrazole (14) to COX-2.** **a** Raw ITC data obtained upon titration of compound **14** with COX-2 protein at room temperature (298.15 K); **b** Plot of the integrated heat signal as a function of the molar ratio of ligand to the protein; **c** Thermodynamic parameters for the binding of compound **14** with COX-2 (*n* = 3, ±s.e.m.)

The success of identifying 5-azido-pyrazole (**14**) as suitable building block for in situ click chemistry reaction prompted us to determine the protein–ligand-binding stoichiometry (*N*), change in thermodynamic parameters, and association constant (*K*_a_) for the binding of 5-azido-pyrazole (**14**) with pure COX-2 protein under physiological conditions using ITC. The determined binding stoichiometry for compound **14** (*N* = 1.02 ± 0.0259) indicated that one molecule of the 5-azido-pyrazole (**14**) was bound per COX-2 isozyme. The high association constant (*K*_a_ = 2.20 × 10^−6^ M^−1^) and favourable free energy change (ΔG < 0; ΔG = −36.20 kJ mol^−1^) clearly supports the assumption of tight binding of compound **14** to the COX-2 protein (Supplementary Table 1). The ITC results summarized in Fig. 5 demonstrate that protein–ligand interactions are thermodynamically favored and that they are mainly driven by the enthalpy factor. The binding of compound **14** to COX-2 isozyme involves an exothermic reaction (ΔH < 0; ΔH = −34.51 kJ mol^−1^) which is indicative of a combination of H-bonding, *π*-*π* stacking and ion–dipole interactions. Interestingly, docking studies have also supported the contribution of H-bonding in protein–ligand binding. The determined favorable small entropic change (TΔS > 0; TΔS = 1.69 kJ mol^−1^) also reflects the little contributions of electrostatic contact, conformational freedom, and solvent reorganization.

### In vitro COX-1/COX-2 inhibition assay

All representative triazole compounds (**7–12**, **16–25, 28**, **29**, **32,** and **33**) as well as 5-azido-pyrazole click chemistry building blocks (**5**, **14**, **27,** and **31**) were tested for in vitro COX-1/COX-2 inhibitory activity. All compounds displayed higher inhibitory potencies for the COX-2 isozyme (IC_50_ range: 0.05 to 26.0 µM) compared to the COX-1 isozyme (IC_50_ range: 45.5 to >100 µM) (Table 2). Interestingly, both in situ click chemistry hit compounds (**18**, **21**) showed particularly high COX-2 inhibitory potency (COX-2 IC_50_ = 0.09 µM and 0.05 µM, respectively) and COX-2 selectivity (COX-2 selectivity index (SI) = 1,111 and 2,000, respectively). Moreover, compound **21** exhibited better COX-2 inhibitory activity and SI than widely used COX-2 inhibitor celecoxib **1** (COX-2 IC_50_ = 0.07 µM, SI = 1,428).

**Table 2:** In vitro COX-1 and COX-2 inhibition data, COX-2 selectivity index (SI), molecular volume, and calculated log ***P*** data of compounds **1**, **5**, **14**, **7–12**, **16–25**, **28, 29**, **32,** and **33**


Compound	*R* ^1^	X	COX-2 IC_50_ (µM)	COX-1 IC_50_(µM)	COX-2 SI	Cell COX-2 IC_50_ (µM)	***V***_molecular_ [Å^3^]	log ***P***
**7**	H	H	0.9	>100	>111.1	3.0	327.9	6.03
**8**	Cl	H	0.16	>100	>625	4.0	341.4	6.58
**9**	F	H	0.14	>100	>714.2	2.0	332.8	6.18
**10**	OCH_3_	H	15.2	>100	>6.5	–	353.4	5.90
**11**	CH_3_	H	3.2	>100	>31.2	2.0	344.5	6.51
**12**	CH_2_CH_3_	H	18	>100	>5.5	–	361.3	6.93
**16**	H	SO_2_CH_3_	10	>100	>10	–	321.0	3.34
**17**	Cl	SO_2_CH_3_	1.1	>100	>90.9	1.0	334.6	3.89
**18**	F	SO_2_CH_3_	0.09	>100	>1,111.1	0.06	326.0	3.49
**19**	OCH_3_	SO_2_CH_3_	26	>100	>3.84	–	346.6	3.21
**20**	CH_3_	SO_2_CH_3_	19	>100	>5.26	–	337.6	3.82
**21**	NH_2_	SO_2_CH_3_	0.05	>100	>2,000	0.08	332.3	2.53
**22**	CF_3_	SO_2_CH_3_	0.22	>100	>454.5	0.15	352.3	4.26
**23**	–	SO_2_CH_3_	6.2	>100	>16.1	–	332.6	3.18
**24**	–	SO_2_CH_3_	0.82	>100	>121.9	0.7	344.6	1.20
**25**	–	SO_2_CH_3_	5.2	>100	>19.2	11.0	352.4	2.34
**28**	F	SO_2_CH_3_	0.14	>100	>714.2	0.2	380.8	4.89
**29**	NH_2_	SO_2_CH_3_	2.7	>100	>37.0	0.1	387.2	3.93
**32**	F	H	12.0	45.5	3.7	0.1	278.0	4.79
**33**	NH_2_	H	8.6	57.9	6.73	0.1	284.3	3.83
**5**	–	H	18.1	>100	>5.5	>1.0	232.9	–
**14**	–	SO_2_CH_3_	4.4	>100	>22.7	10.0	226.1	–
**1**	Celecoxib		0.07	>100	>1428	0.09	298.6	4.34

Assays were conducted as described in the Methods section. IC_50_, half-maximal inhibitory concentration; SI, In vitro COX-2 selectivity index: [(COX-1 IC_50_)/(COX-2 IC_50_)]; Compounds were tested in HCA-7 cells; data are the mean of three determinations; —, not tested. *V*_molecular_, molecular volume, after energy minimization with the molecular mechanic’s geometry optimization module, the molecular volume was calculated using the Alchemy 2000 program (Tripos Inc.). The log *P* value was calculated using ChemDraw Professional 15.0.0.106.

The relative inhibitory potency and selectivity profile were observed in the following order: **21** (R^1^ = NH_2_), >**9**, **18** (R^1^ = F) >**8**, **17** (R^1^ = Cl), which shows that the *para* substituent (R^1^) on the C-1 phenyl ring of triazole is crucial for both COX-2 inhibitory potency and high COX-2 selectivity. However, compounds **23–25** containing heterocyclic or aliphatic (ibuprofen like) motifs on C1 position of triazole ring displayed only low inhibitory potencies towards COX-2. Comparison of COX-2 inhibitory potency profiles of compounds **28, 29, 32,** and **33** with **7–12** and **16–25** confirmed the importance of the SO_2_CH_3_ COX-2 pharmacophore as an important structural requirement for high COX-2 inhibition. Among all tested compounds, triazoles **18** and **21** were identified as the most potent and selective COX-2 inhibitors. Thus, obtained in vitro COX-1/2 enzyme inhibitory data evidently demonstrate that in situ click chemistry reaction can also be employed to identify highly potent and selective COX-2 inhibitors. However, compounds **8** (COX-2 IC_50_ = 0.16 µM), **9** (COX-2 IC_50_ = 0.14 µM), and **22** (COX-2 IC_50_ = 0.22 µM) also showed appreciable COX-2 inhibitory potencies in the in vitro COX-binding assay albeit nor formation of compounds **8**, **9,** and **22** was detected in the COX-2 mediated in situ click chemistry reaction. Based on the in vitro COX-2 inhibitory data, seventeen compounds (Table 2) were selected and further evaluated for their cellular COX-2 inhibitory activity in COX-2 expressing colorectal cancer cell line HCA-7. Both in situ click chemistry hit compounds (**18**, **21**) also displayed highest cell-based COX-2 inhibitory potencies. Compounds **18** and **21** displayed a better COX-2 inhibitory profile in HCA-7 cells (COX-2 IC_50_ = 0.06 µM and 0.08 µM, respectively) compared to celecoxib (COX-2 IC_50_ = 0.09 µM).

### In vivo anti-inflammatory activity

**Fig. 6:**
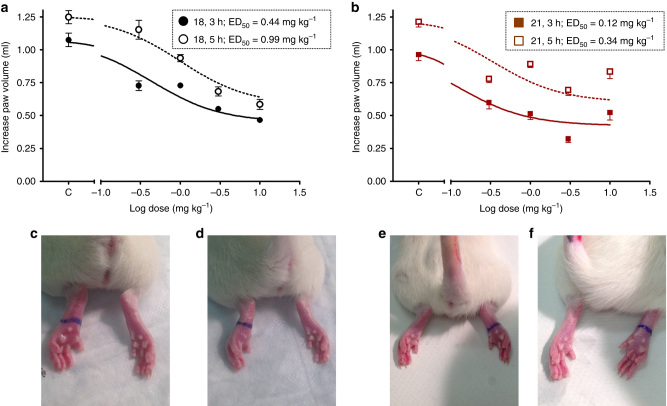
**In vivo anti-inflammatory activity profile of in situ click chemistry hits.** **a**, **b** Effective dose (ED_50_) calculated at 3 and 5 h time after treatment of compound **18** (*n* = 4, ±s.e.m.) and **21** (*n* = 5, ±s.e.m.); **c**, **e** Control; **d**, **f** Images showing the in vivo effect at 3 h after treatment with 0.3 mg kg^−1^ p.o. of compounds **18** (left paw) and **21** (right paw), respectively

The two in situ click chemistry hit compounds **18** and **21** were further studied for their in vivo anti-inflammatory potency. Both compounds proved to be highly potent anti-inflammatory agents. ED_50_ values were determined in a carrageenan-induced rat paw edema assay at 3 and 5 h time points after treatment with 0.3, 1.0, 3.0, and 10 mg kg^−1^ p.o. of compounds **18** and **21** (Fig. 6, Supplementary Figs 4 and 5). Compound **18** showed high anti-inflammatory activity at 3 h (ED_50_ = 0.44 mg kg^−1^) and 5 h (ED_50_ = 0.99 mg kg^−1^) time points. An ever-higher anti-inflammatory activity at 3 h (ED_50_ = 0.12 mg kg^−1^) and 5 h (ED_50_ = 0.34 mg kg^−1^) time points was found for compound **21**. However, both compounds were found to be >25 times more potent than widely used COX-2 inhibitor celecoxib (ED_50_ = 10.8 mg kg^−1^ at 3 h). To the best of our knowledge, compounds **18** and **21** are the most potent known anti-inflammatory agents. The determined very high COX-2 inhibitory potency and selectivity of in situ click chemistry hit compounds **18** and **21** clearly demonstrates the power of innovative in situ click chemistry for the identification of novel highly potent and selective COX-2 inhibitors.

### Molecular docking studies

**Fig. 7:**
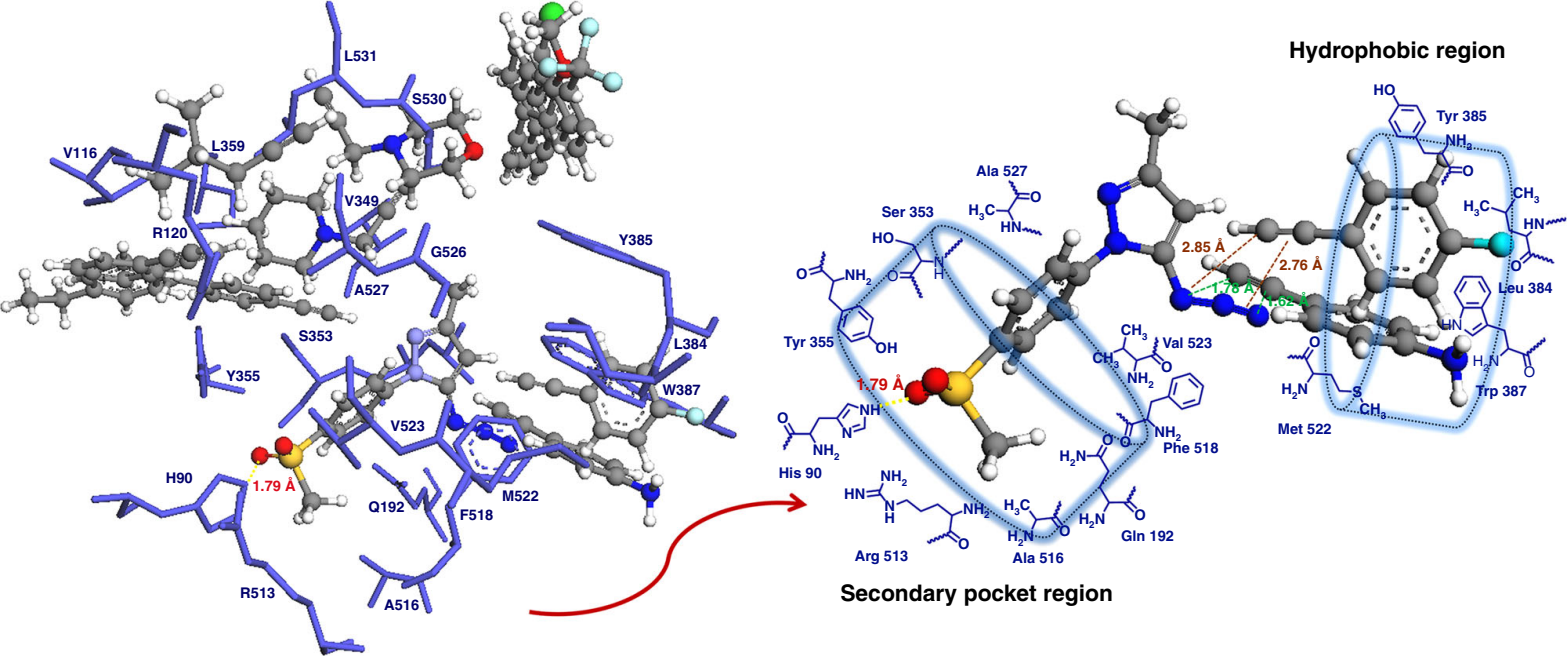
**Computational analysis of click chemistry building blocks in the COX-2 active site.** Molecular docking of 5-azido-pyrazole compounds (**5**, **14**, **27,** and **31**) and alkynes (**6a–6f** and **15a–15e**) in the binding site of COX-2 isozyme (PDB ID: 6COX). Hydrogen atoms of amino-acid residues are omitted for clarity

To further support our experimental results and to investigate the mode of ligand–protein molecular interactions, all click chemistry building blocks (**6, 14, 27, 31, 6a–6f,** and **15a–15e**) and final triazole compounds (**7–12, 16–25, 28, 29, 32,** and **33**) were docked into the COX-2 active site. It was found that the *para* SO_2_CH_3_ group in 5-azido-pyrazole compound (**14**) was deeply inserted into the secondary-binding pocket region of the COX-2 active site (Fig. 7 and Supplementary Fig. 6). The *O*-atoms of the SO_2_CH_3_ group showed H-bonding interaction with one of the terminal amino group of R513 (distance O---NH_2_ = 2.25 Å) and with the nitrogen atom of the imidazole ring of the H90 residue (distance O---N = 1.92 Å). Interestingly, the other 5-azido-pyrazoles (**5, 27,** and **31**) were located away from the secondary pocket region, and did not indicate any H-bonding interactions (Supplementary Fig. 7), except for compound **27** where one of the *O*-atoms of the SO_2_CH_3_ group was H-bonded to the-NH_2_ group of R120 (distance O---NH_2_ = 1.96 Å). Alkynes possessing either a-F or -NH_2_ group (**6c** and **15a**) were positioned in the vicinity of azide group of compound **14** (Fig. 5 and Supplementary Fig. 9), whereas the other three alkynes (**6b**, *R* ^1^ = Cl; **6d**, *R* ^1^ = OCH_3_; and **15b** *R* ^1^ = CF_3_) were clustered together near Y385; alkynes with *para* CH_3_ or *para* C_2_H_5_ groups and heterocyclic rings (**6e, 6f,** and **15c**–**15e**) were also docked far away from the azide group of pyrazole compounds (**6,14, 27,** and **31**). Molecular docking indicated that 5-azido-pyrazole (**14**) and alkynes (**6c** and **15a**) were favorably oriented in such a way that the azide group (-N_3_) is present in proximity of 4-ethynyl group (-C≡CH) of both alkynes; and the calculated distance between the terminal carbon atom of the ethynyl groups in **6c** (*R* ^1^ = F) and **15a** (*R* ^1^ = NH_2_) and one of the nitrogen atoms of the azide group in compound **14** was 1.78 and 2.85 Å, respectively (Fig. 7). Therefore, the observed formation of in situ click chemistry products (**18** and **21**) can be attributed to the close and special orientation of reactive functional groups of the complementary click chemistry building blocks (**14**, **6c,** and **15a**) in the COX-2-binding site.

**Fig. 8:**
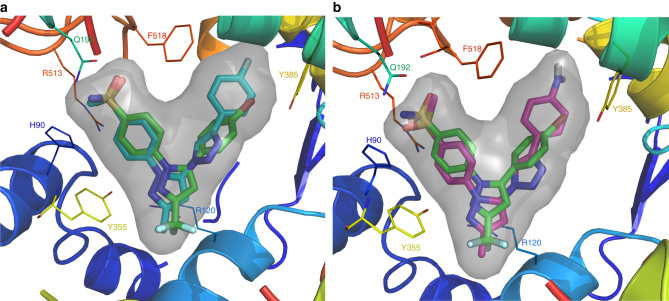
**Comparison of the binding conformation of in situ click chemistry reaction hit compounds with crystalized SC558.** **a** In COX-2 isozyme (PDB:6COX) the compound **18** (*cyan* carbon) and SC558 (*green* carbon) are displayed in stick mode and surface of compound **18** is highlighted (*grey*) color; **b** compound **21** (*magenta* carbon) and SC558 (*green* carbon) are displayed in stick mode and surface of compound **21** is highlighted (*grey*) color

Further molecular docking studies with in situ click chemistry products (**18**, **21**) demonstrated that both compounds fit favorably into the COX-2-binding sites (*E*_intermolecular_ = −15.9 and −16.8 kcal mol^−1^ respectively) displaying favorable electrostatic interactions with the secondary pocket residues (Supplementary Figs 8 and 9). For compound **18**, the *O*-atoms of the *para* SO_2_CH_3_ group showed H-bonding with R513 (distance O---N = 2.90 Å) and the H90 residue (distance O---N = 2.37 Å); and two of the N-atoms of the pyrazole ring were also H-bonded to A527 (Supplementary Fig. 9). In the case of compound **21,** the *O*-atoms of the *para* SO_2_CH_3_ group indicated H-bonding with Y355 (distance O---HO = 2.39 Å) which is considered as one of the important catalytic amino acid for COX reaction of enzyme^57^, with the R513 residue (distance O---N = 2.08 Å); and the NH_2_ group in compound **21** showed H-bonding with Q192 (distance N---O = 2.64 Å) in the COX-2 active site (Supplementary Fig. 9). In contrast, upon docking of all azide compounds (**5**, **14, 27,** and **31**) and alkynes (**6a–6f** and **15a–15e**) into the COX-1 active site, it was found that none of the azide compounds were located near any of the alkyne compounds, indicating less opportunity for an in situ click chemistry reaction in the COX-1 active site. The preference of the COX-2 isozyme for in situ click chemistry reaction over the COX-1 isozyme can be explained by considering the structural differences of both enzymes. The size of the COX-2 isozyme active site (volume = 394 Å^3^) is about 25% larger than the COX-1 isozyme-binding site (volume = 316 Å^3^). Moreover, the COX-2 isozyme possesses an additional secondary pocket region. Because of these two distinct structural differences, the COX-2 isozyme can conjointly hold two reactive/more bulky ligands more appropriately than the COX-1 isozyme. The energy of intermolecular interactions (*E*_intermolecular_) between the ligand and the enzyme residues obtained after computational studies (docking) for all compounds within the COX-1 and COX-2 active site are summarized in Supplementary Table 2 and Supplementary Fig. 8. Interestingly, compound **18** and **21** occupy an orientation that is very similar to the binding mode of crystallized COX-2 inhibitor SC558 in the COX-2 isozyme (Fig. 8). Overall, the computational results suggest that similar to the orientation of parent azide click chemistry building block **14**, the 4-methanesulfonyl-phenyl motif of in situ click chemistry hit compounds **18** and **21** occupy conformational spaces in the secondary-binding pocket region of the COX-2-binding site, and the fluoro/amino-phenyl ring portion is embedded into the hydrophobic pocket; H90, R120, Q192, Y355, Y 385, R513, and F518 are the key amino-acid residues, which considerably contributed in the strong protein–ligand interactions. All these observations agree with the experimental results and support the success of in situ click generation of **18** and **21** from clickable building blocks **14** (azide) and **6c**/**15a** (alkyne) in the COX-2-binding pocket.

## Discussion

We have, for the first time, demonstrated the use of in situ click chemistry for the identification and synthesis of highly potent and selective COX-2 isozyme inhibitors. In situ click chemistry with COX-2 isozyme as molecular template was highly regioselective and products are exclusively formed as 1,4-regioisomers through[3,2]-cycloaddition reaction inside the COX-2-binding pocket. Our comprehensive SAR and computational studies suggest that size, presence of a SO_2_Me COX-2 pharmacophore, proper orientation of the azide click chemistry building block inside the COX-2-binding site, H-bonding, and hydrophobic interactions of building blocks collectively contribute to the in situ click chemistry synthesis of highly potent and selective COX-2 isozyme inhibitors. Moreover, we demonstrated that the COX-2 isozyme is capable of finding and selecting the most favourable complementary azide and alkyne building block pairs from a pool of reactive precursors to generate its own ‘best fit’ inhibitor. Results of this study provide an efficient tool for economical and fast screening of potential drug candidates. Based on the extremely high in vivo activity of identified and characterized in situ click chemistry hit compounds, we are currently working to expand this strategy for the detection of COX-2 expression at the cellular and in vivo level.

## Methods

### Chemical synthesis

Detailed procedures for the synthesis of all new compounds and their characterization are provided in the Supplementary Methods (For ^1^H and^13^C spectra of compounds **14**, **18,** and **21**, see Supplementary Figs 10, 11, 12, 13, 14, and 15).

### In situ click chemistry screening procedure for binary reagent mixtures

To investigate the COX-2 isozyme templated synthesis, each 5-azido-pyraozle (**5, 14, 27,** and **31,** 1 µl of 3 mM DMSO solution) and alkyne (**6a**–**6f, 15a**–**15e**, 1 µl of 20 mM DMSO solution) were pairwise mixed with human recombinant COX-2 isozyme (95 µl COX-2) in 1 µl of 1 M Tris-HCl, pH 8.0. The each reaction mixture was vortexed for 1 min, and then incubated at room temperature (For temperature dependency of COX-2 enzyme activity, see Supplementary Fig. 16). Final reagent concentrations were as follows: COX-2 (7 µM), azide (30 µM) alkyne (200 µM). After 3, 6, 9, 12, 15, 18, 21, and 24 h each sample was analyzed in triplicate by injecting (10 µl) into the LC/MS instrument with SIM mode (Water’s Micromass ZQTM 4000 LC−MS instrument, operating in the ESI-positive mode, equipped with a Water’s 2795 separation module). Calibration curve for hit compounds **18** and **21** is given in Supplementary Fig. 17. Summaries of all LC/MS data are presented in Supplementary Tables 3–7. Separations were performed in triplicate using a Kromasil 100-5-C18 (100 μm pore size, 5 μm particle size) reverse phase column (2.1 mm diameter × 50 mm length), preceded by a Kromasil 100-5-C18 2.1 × guard column. Separations were effected using a gradient MeCN/H_2_O (0.05% trifluoroacetic acid (TFA))/MeOH in 40/30/30, v/v/v over 15 min at flow rate 0.25 ml min^−1^. Operating parameters were as follows: capillary voltage = 3.5 kV; cone voltage = 20 V; source temperature = 140 °C; sesolvation temperature = 250 °C; cone nitrogen gas flow = 100 l h^−1^; desolvation nitrogen gas flow = 550 l h^−1^. The identities of triazole products (retention time of 6.73 min for **18**), (retention time of 4.56 min for **21**), and the internal standard (retention time of 10.89 min) were confirmed by molecular weight and comparison of the retention times of the authentic products formed from copper catalyzed reactions. Control experiments in the presence of BSA (1 mg mL^−1^) instead of the COX-2 enzyme as well as in the absence of COX-2 enzyme and the known COX-2 selective inhibitor (1 µl of celecoxib, 100 µM final concentration) were run as described above. For multicomponent in situ click chemistry reactions, each azide (**5, 14, 27,** and **31,** 1 µL of 3 mM DMSO solution) and eleven alkynes (**6a**–**6f** and **15a**–**15e,** 1 µl of 20 mM DMSO solution) were thoroughly mixed together in the presence of COX-2 isozyme (95 µl COX-2) in 1 µl of 1 M Tris-HCl, pH 8.0 and incubated at room temperature. After 24 h each sample was analyzed in triplicate by injecting (10 µl) into the LC/MS instrument by following the procedure described above, except the ions are monitored for all possible masses. The cyclo addition products were identified by their molecular weights and by comparison of the retention times of authentic products prepared through Cu-catalyzed reactions. Control experiments using BSA (1 mg ml^−1^) in place of COX-2 isozyme and in the absence of COX-2 isozyme were run consecutively.

### Isothermal titration calorimetry

The thermodynamics of the precursor 5-azido-pyrazole (**14**) binding with pure human COX-2 protein studied with a high sensitivity ITC instrument (VP-ITC, MicroCal). All titrations were performed by following VP-ITC general procedure described in manual. Protein (20 µM) and ligand (200 µM) solution were prepared in PBS buffer, pH 7.4. Prior to each ITC run, all solutions were filtered using membrane filters (pore size 0.45 μm) and thoroughly stirred and degassed using Thermovac accessory for 20 min to remove any air bubbles at 25 °C. In a typical experiment, aliquots (6 µl) of ligand solution (prepared at 10 times the COX-2 protein concentration) were injected with a computer-controlled stirrer-syringe into a reaction cell containing COX-2 protein. Titrations were carried out with a stirring speed of 400 rpm and 240 s intervals between injections. All experiments were conducted at 25 °C. Control experiments, including titration of the ligand into buffer alone or COX-2 protein into buffer alone, were carried to determine the heat due to dilution and subtracting during the original experiment. Calorimetric data analysis was performed with ORIGIN software provided by MicroCal. All results, including the binding constant (*K*_a_), stoichiometry of binding (*N*), and thermodynamics of binding (Δ*H* and TΔ*S*) were determined by fitting the experimental binding isotherms. Each ITC experiment was repeated three times and showed good reproducibility.

### COX inhibition assay

The ability of known COX-2 selective inhibitor celecoxib (**1**), 5-azido-pyraozles (**5** and **14**) and new triazole products (**7–12**, **16–25, 28**, **29**, **32,** and **33**) to inhibit ovine COX-1 and recombinant human COX-2 was determined using a COX inhibitor assay (Cayman Chemical, Ann Arbor, USA; item number: 700100) following the manufacturer’s protocol. Each compound was assayed in concentration range of 10^−9^ M to 10^−3^ M, in triplicate. PRISM5 software was used to calculate IC_50_ values. In addition to celecoxib, both Dup-697 (potent COX-2 inhibitor) and SC-560 (potent COX-1 inhibitor) were used as internal controls during screening test compounds.

### In vivo anti-inflammatory activity

The two lead compounds **18** and **21** were advanced for in vivo anti-inflammatory activity study and celecoxib (**1**) was used as reference drug. In vivo anti-inflammatory activity was measured using a carrageenan-induced rat paw edema assay. In brief, three to five male Sprague–Dawley rats, 8–11-weeks-old, weighing 180–200 g (Charles-River Canada) were used in each group. Animals were randomized into different treatment groups based on similar paw size and body weight. Test compounds **18** and **21** suspended in water containing 1% methyl cellulose were administered orally for a minimum of four different doses (0.3. 1, 5, 10 mg kg^−1^) 1 h prior to a 0.05 ml subcutaneous injection of fresh 1% carrageenan in 0.9% NaCl solution under the plantar skin of the hind paw. Control experiments were identical, except that the vehicle did not contain a test compound. The volume of the injected paw was measured at 0, 3, and 5 h using a UGO Basile 7141 Plethysmometer (series no. 43201), each value is mean of 10 measurements. A dose–response curve was constructed using GraphPad Prism 5.0 and ED_50_were calculated. No unusual change in behavior and toxic effects was noticed in all animals. In vivo anti-inflammatory assays were carried using a protocol approved by the Health Sciences Animal Welfare Committee, University of Alberta, Edmonton, Canada.

### Molecular docking procedure

The detailed procedure for the molecular docking is provided in the Supplementary Information.

### Statistical analysis

Unless specified, all data were obtained from at least triplicate samples and represent at least three independent experiments and presented as mean ± s.e.m. Graphs were constructed using GraphPad Prism 4.0 (GraphPad Software). Where applicable, statistical differences were tested by unpaired Student’s *t*-test and were considered significant for *P* < 0.05.

### Data availability

The authors declare that all data supporting the findings of this study are available in the article and in the supplementary information file. Additional information are available from the corresponding author upon request.

## Electronic Supplementary Material

Supplementary Information Supplementary Figures, Supplementary Tables, Supplementary Methods and Supplementary References.
