# Supplementary material for: In situ click chemistry generation of cyclooxygenase-2 inhibitors
Source: Nat Commun. 2017 Feb 23;8:1. doi: 10.1038/s41467-016-0009-6 (PMC5431875; doi:10.1038/s41467-016-0009-6)
Supplement: Supplementary file 1 — Supplementary Figures, Supplementary Tables, Supplementary Methods and Supplementary References. [file 41467_2016_9_MOESM1_ESM.pdf]

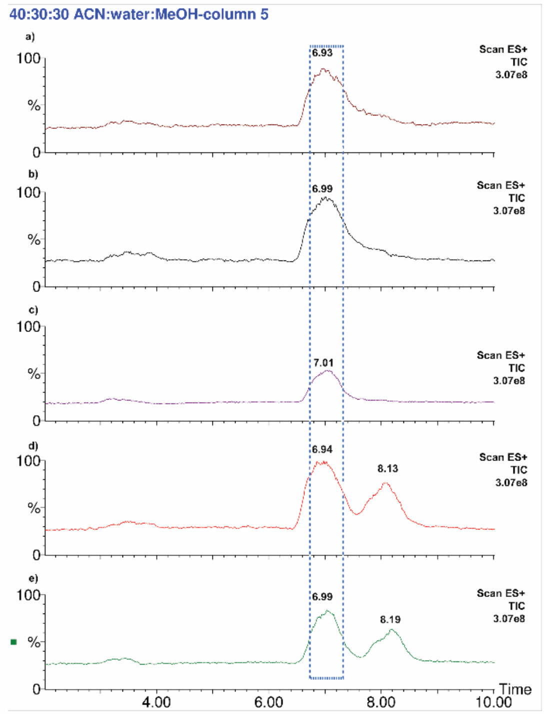

Supplementary Figure 1. Regiochemistry determination for compound 18. Determination of *in situ* click chemistry reaction regiochemistry by LCMS-SIM analysis. a) Pure *anti*-triazole (**18**) prepared by Cu (I) catalyzed reaction between **14** and **6c**; b) *in situ* click chemistry reaction; c) co-injection of *anti*-triazole and *in situ* click chemistry reaction; d) co-injection of mixture of *syn* and *anti*-triazole and *in situ* click chemistry reaction e) mixture of *syn* and *anti*-triazole.

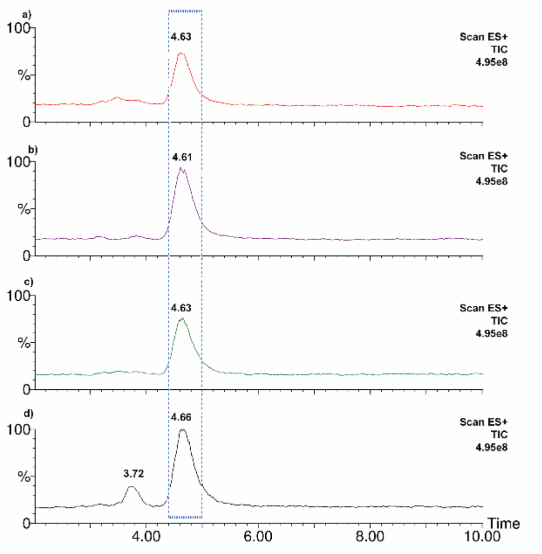

Supplementary Figure 2. Regiochemistry determination for compound 21. Determination of *in situ* click chemistry reaction regiochemistry by LC-MS-SIM analysis. a) Pure *anti*-triazole (**21**) prepared by Cu (I) catalyzed reaction between **14** and **15a**; b) *in situ* click chemistry reaction; c) co-injection of *anti*-triazole and *in situ* click chemistry reaction; e) mixture of *syn* and *anti*-triazole.

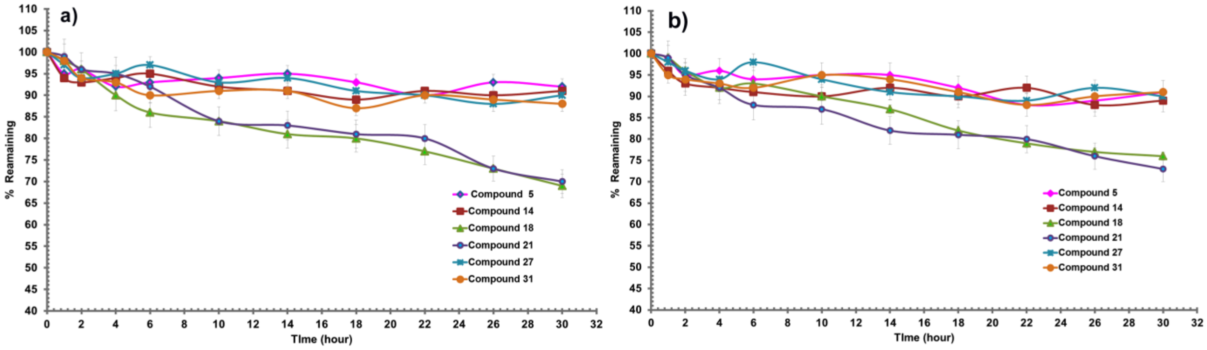

Supplementary Figure 3. Stability in human and rat serum. Stability of compound **5**, **14**, **18**, **21**, **27** and **24** in a) human serum and b) rat serum (n = 3, ±s.e.m.).

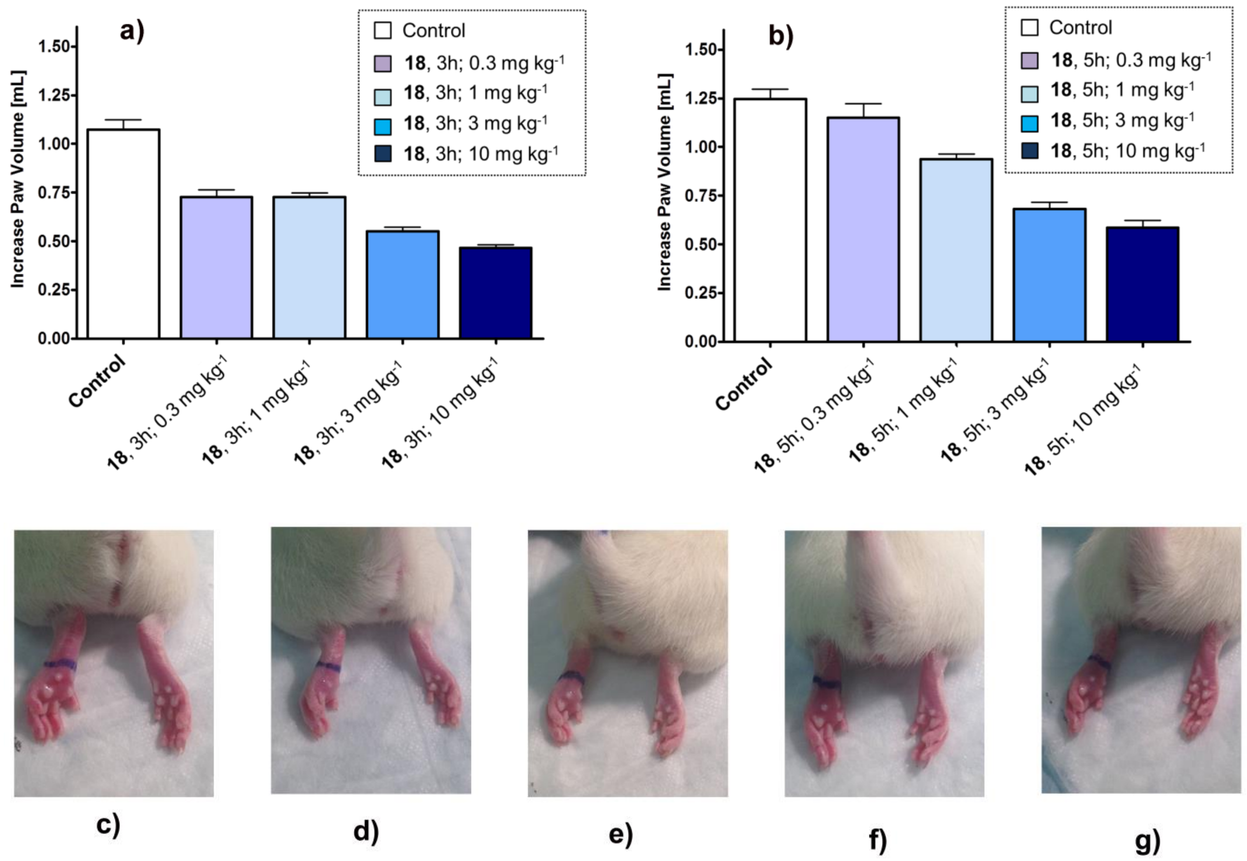

Supplementary Figure 4. *In-vivo* anti-inflammatory activity studies. *In-vivo* anti-inflammatory activity data for compound **18** (n = 4, ±s.e.m.). a) Change in in paw volume at after the treatment with compound **18** at 0.3, 1, 3 and 10 mg kg^{-1} p.o. at 3 h time point; b) change in in paw volume at after the treatment with compound **18** at 0.3, 1, 3 and 10 mg kg^{-1} p.o. at 5 h time point; c) control d)-g) images showing the anti-inflammatory effect of compound **18** at 0.3, 1, 3 and 10 mg kg^{-1} p.o. at 3 h time point.

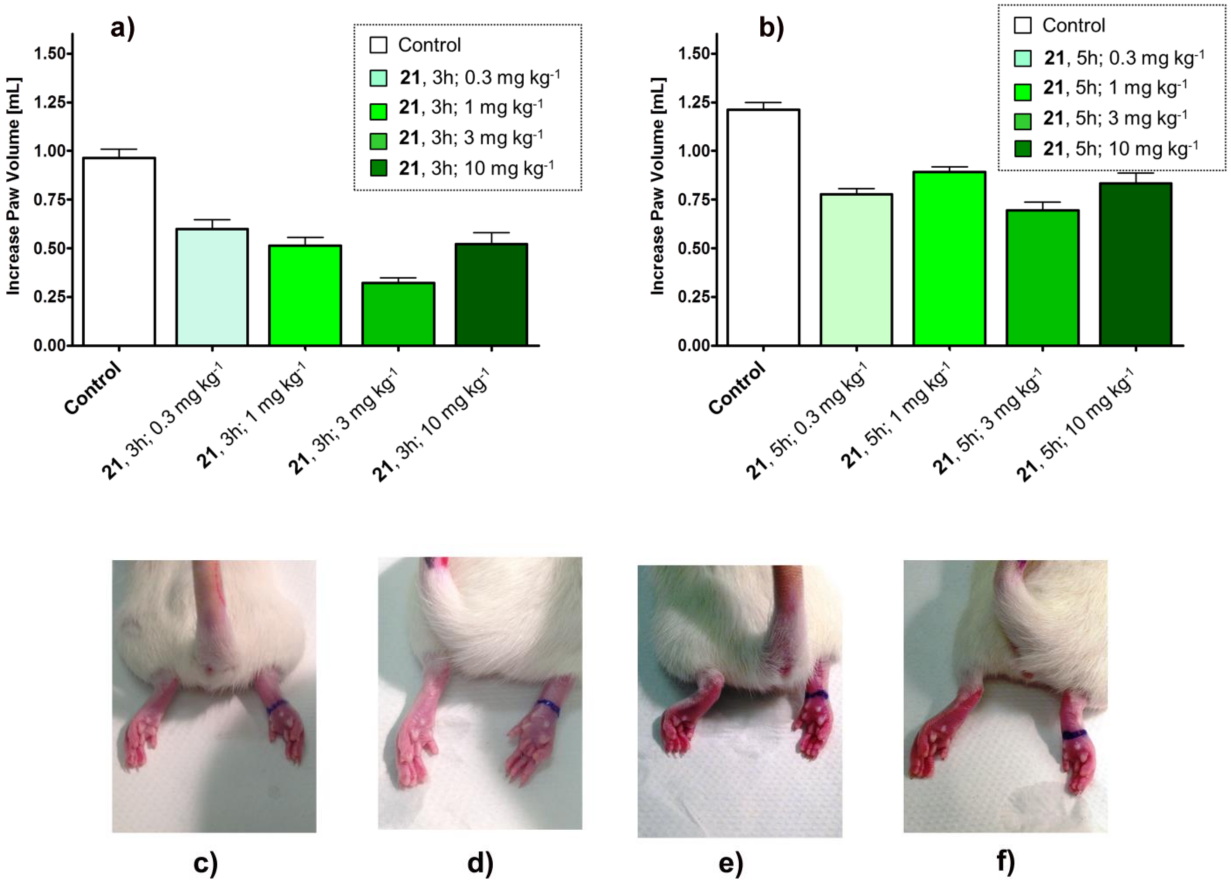

Supplementary Figure 5. ***In-vivo*** **anti-inflammatory activity studies.** *In-vivo* anti-inflammatory activity data for compound **21** (n =5, ±s.e.m.). a) Change in in paw volume at after the treatment with compound **21** at 0.3, 1, 3 and 10 mg kg^{-1} p.o. at 3 h time point; b) change in in paw volume at after the treatment with compound **21** at 0.3, 1, 3 and 10 mg kg^{-1} p.o. at 5 h time point; c) control d)-f) images showing the anti-inflammatory effect of compound **21** at 0.3, 1, and 3 mg kg^{-1} p.o. at 3 h time point.

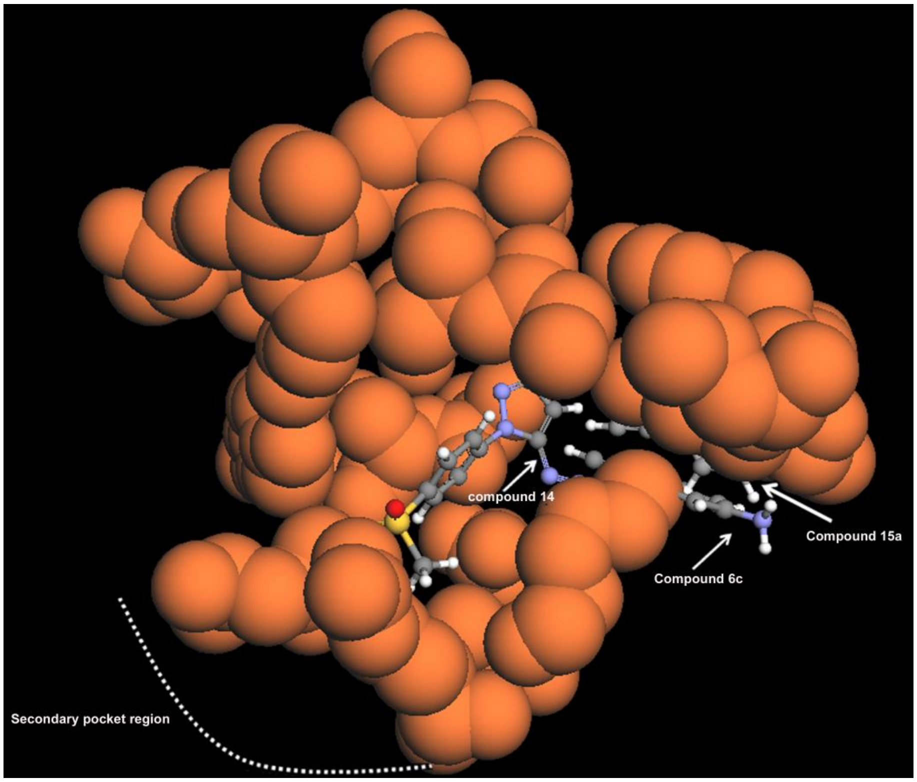

Supplementary Figure 6. Molecular docking results. Molecular docking of 5-azido-pyrazole compound (**14**), alkynes (**6c** and **15a**) in the binding site of COX-2 (PDB ID: 6COX). CPK view to highlight the deep insertion of 5-azido-pyrazole (**14**) into the COX-2 secondary pocket region. Hydrogen atoms of amino acid residues are omitted for clarity.

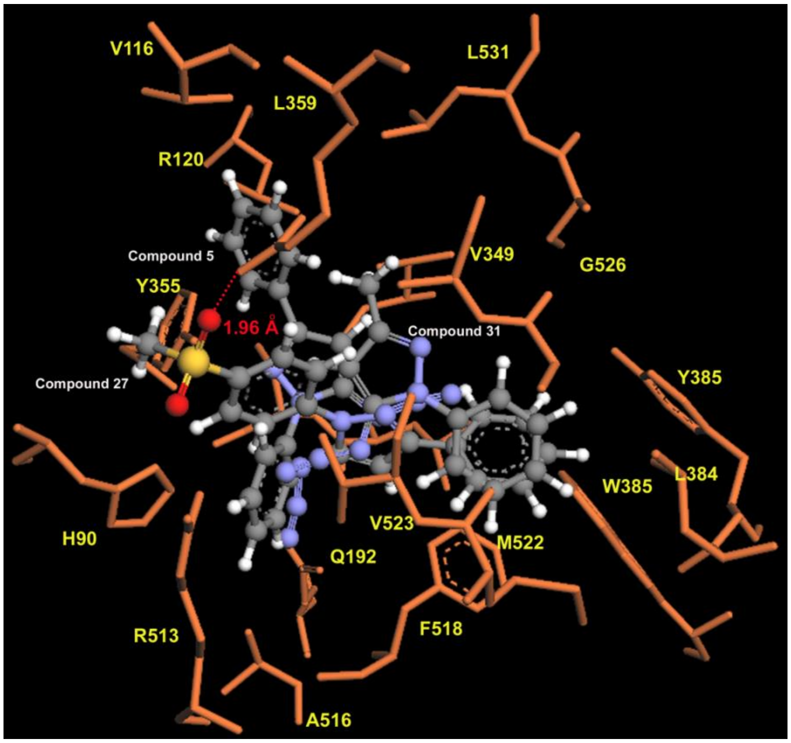

Supplementary Figure 7. Molecular docking results. Molecular docking of 5-azido-pyrazole compounds (**5, 27** and **31**) in the binding site of COX-2 (PDB ID: 6COX). Hydrogen atoms of amino acid residues are omitted for clarity.

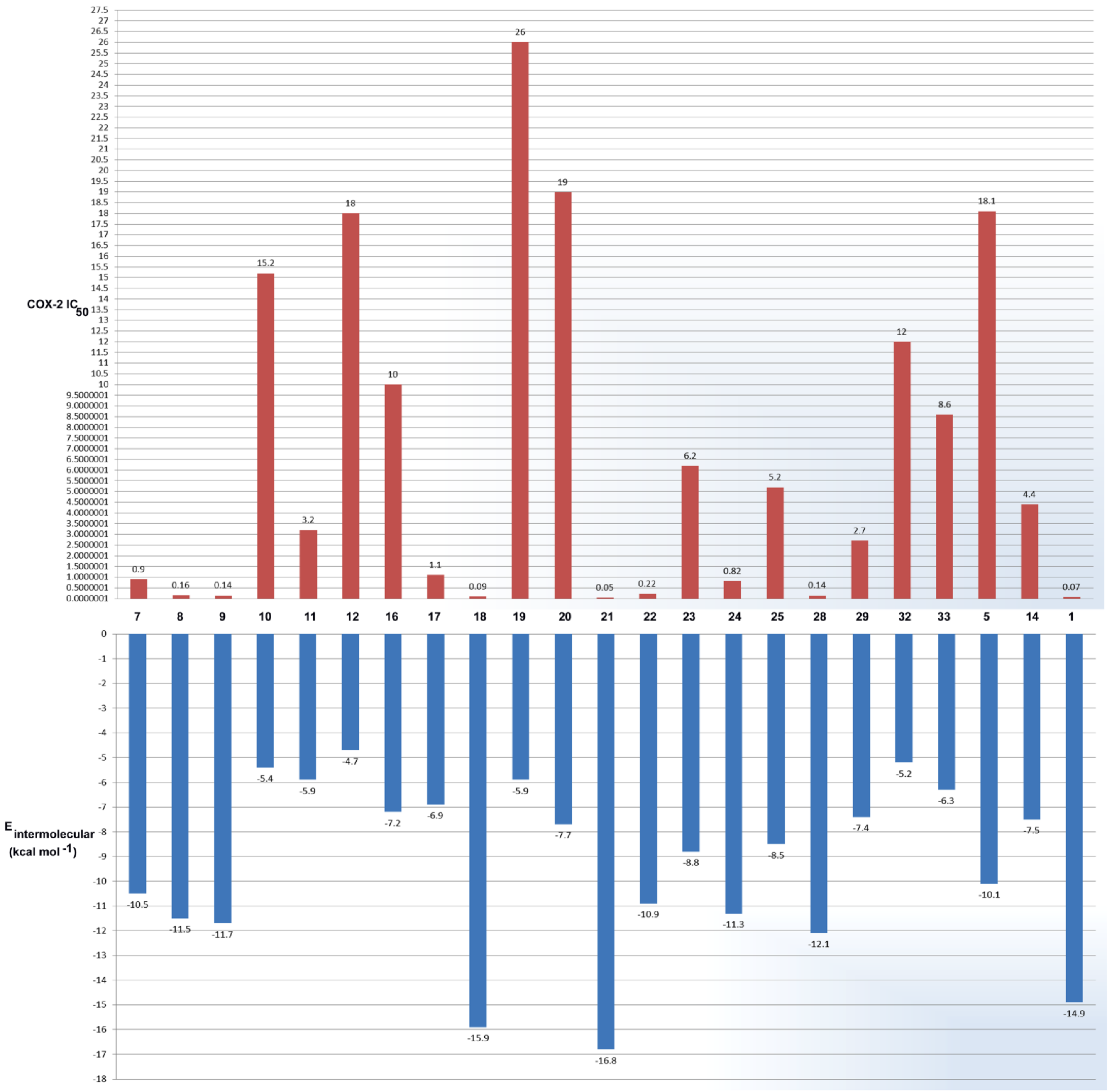

Supplementary Figure 8. Inhibitory activity and energy of intermolecular interactions. Comparison of COX-2 IC_{50} (μM) and energy of intermolecular interactions ($E_{intermolecular}$) between ligand and COX-2 isozyme amino acid residues.

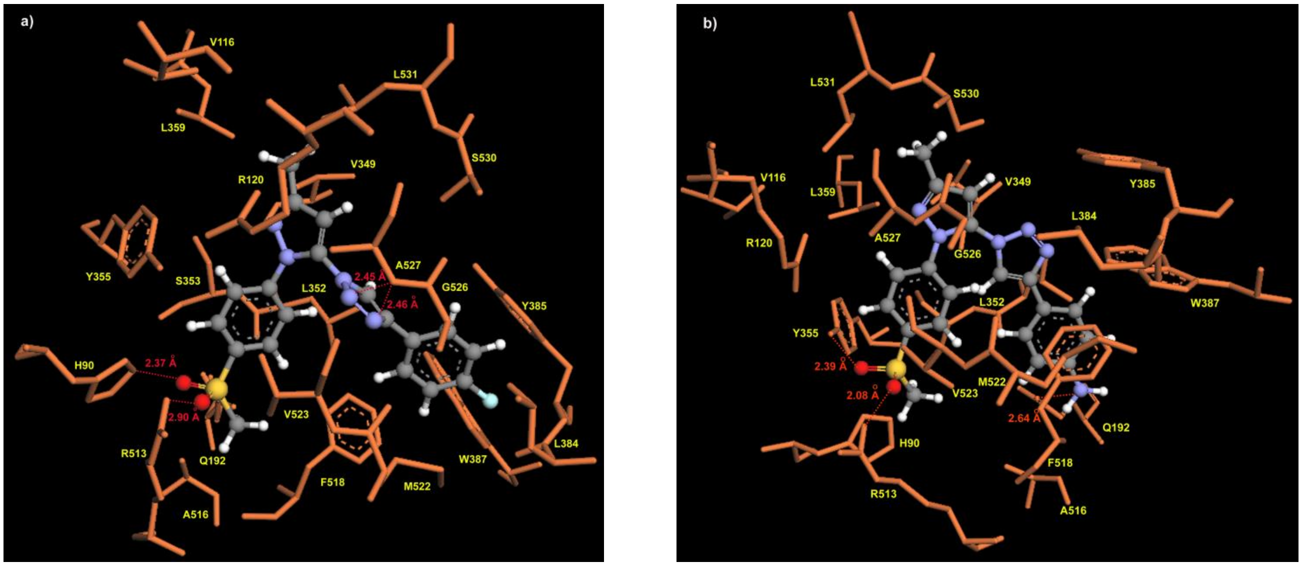

Supplementary Figure 9. Molecular docking in COX-2 enzyme. Molecular docking of compound a) **18** and b) **21** in the binding site of COX-2 (PDB ID: 6COX). Hydrogen atoms of amino acid residues are omitted for clarity.

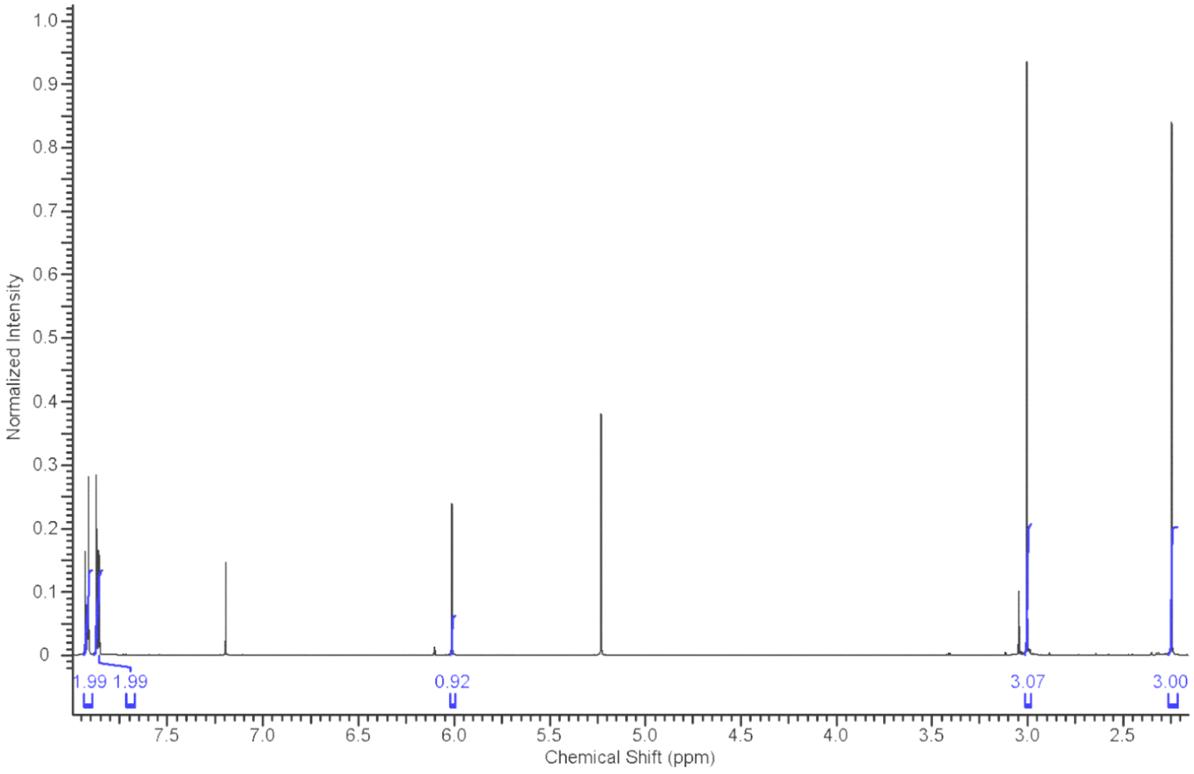

Supplementary Figure 10. ^{1}H NMR spectrum of 5-azido-pyrazole **14**.

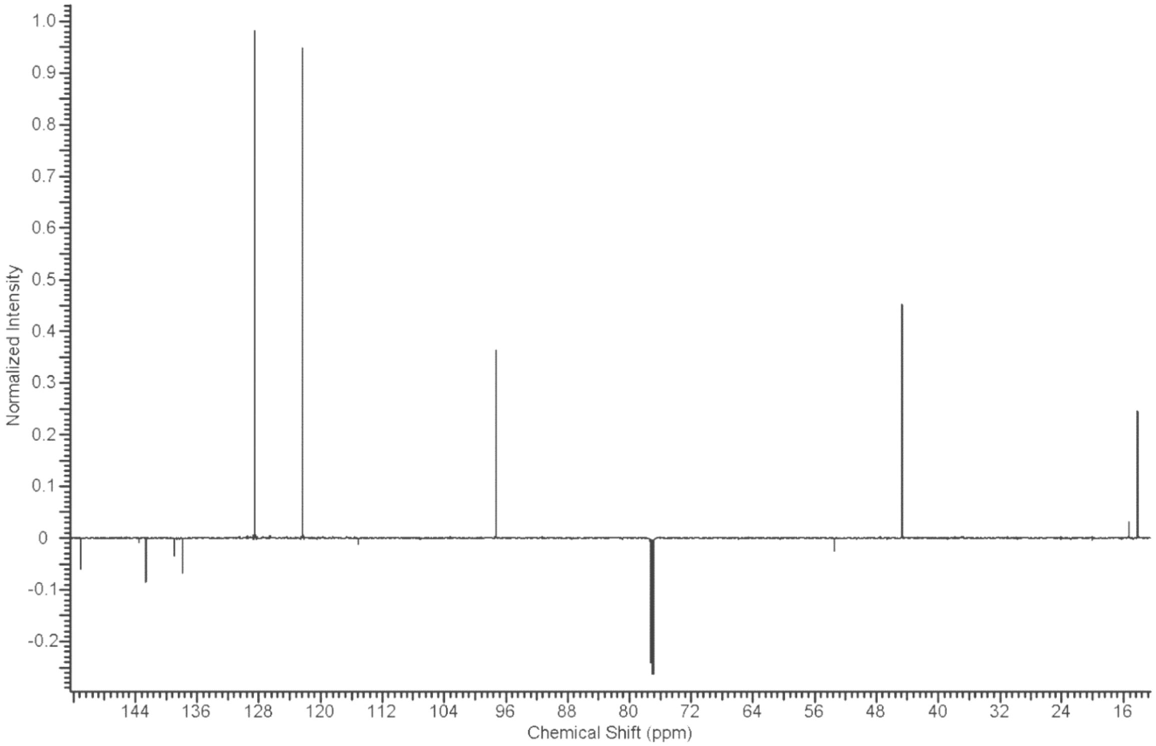

Supplementary Figure 11. ^{13}C NMR spectrum of 5-azido-pyrazole **14**.

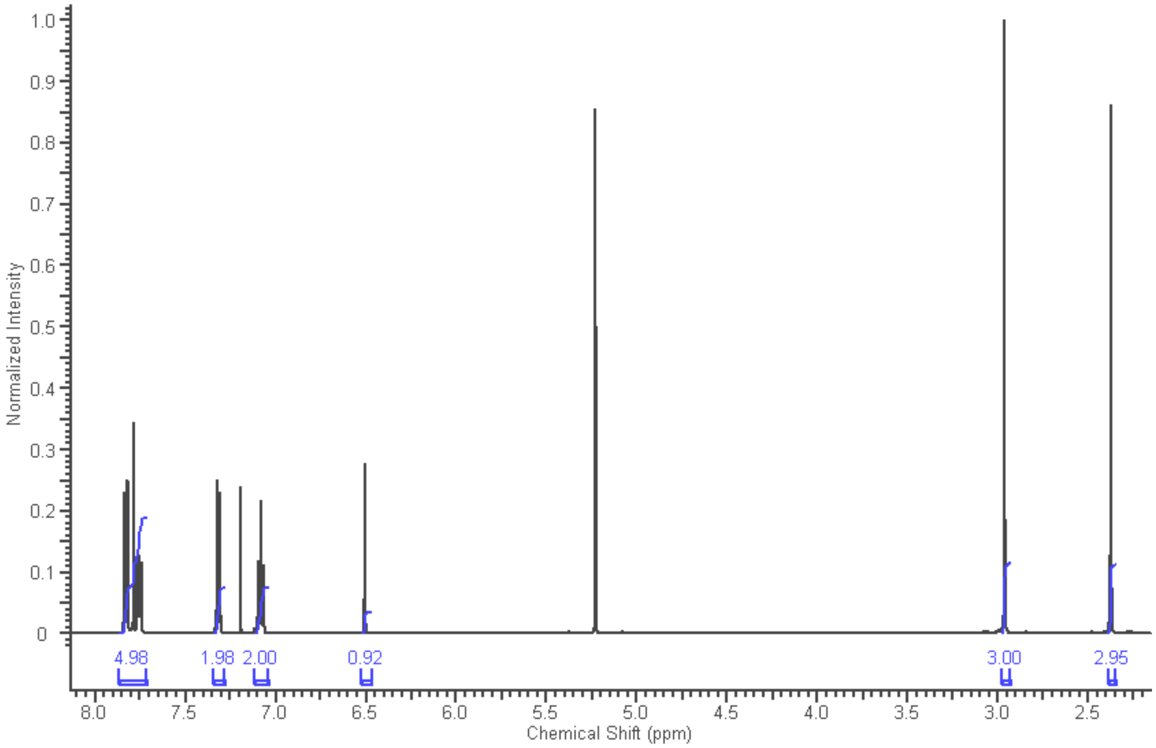

Supplementary Figure 12. ^{1}H NMR spectrum of *in situ* click chemistry hit compound **18**.

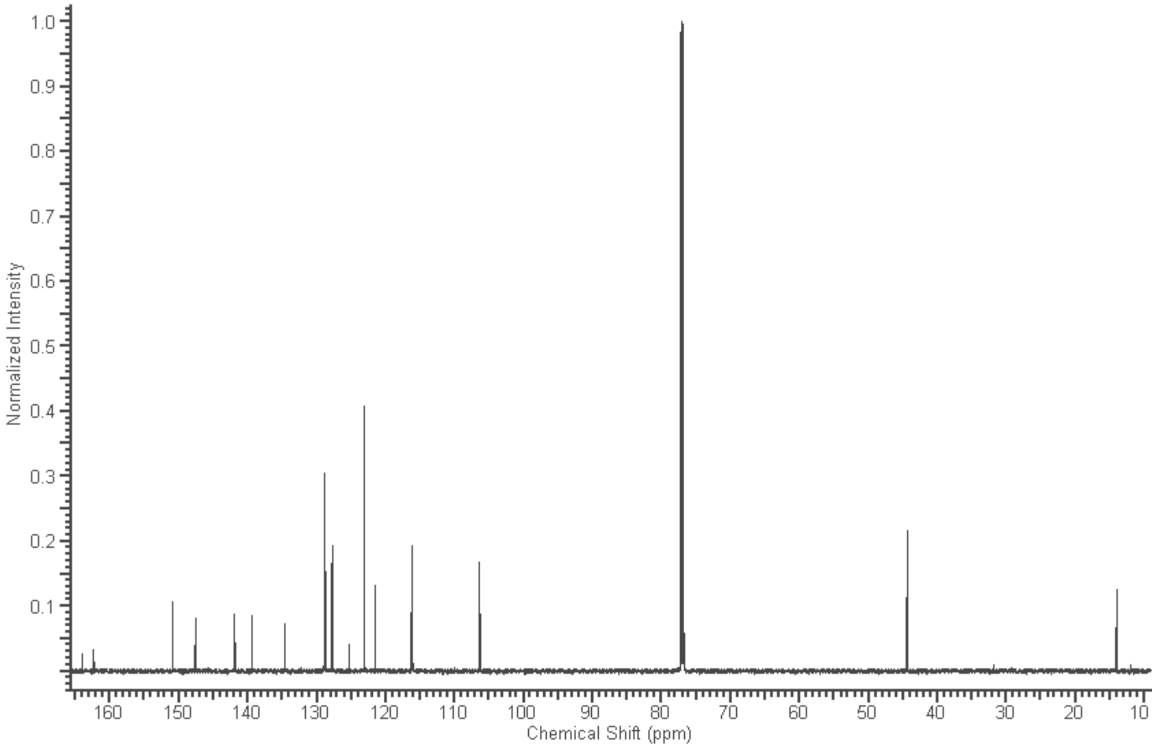

Supplementary Figure 13. ^{13}C NMR spectrum of *in situ* click chemistry hit compound **18**.

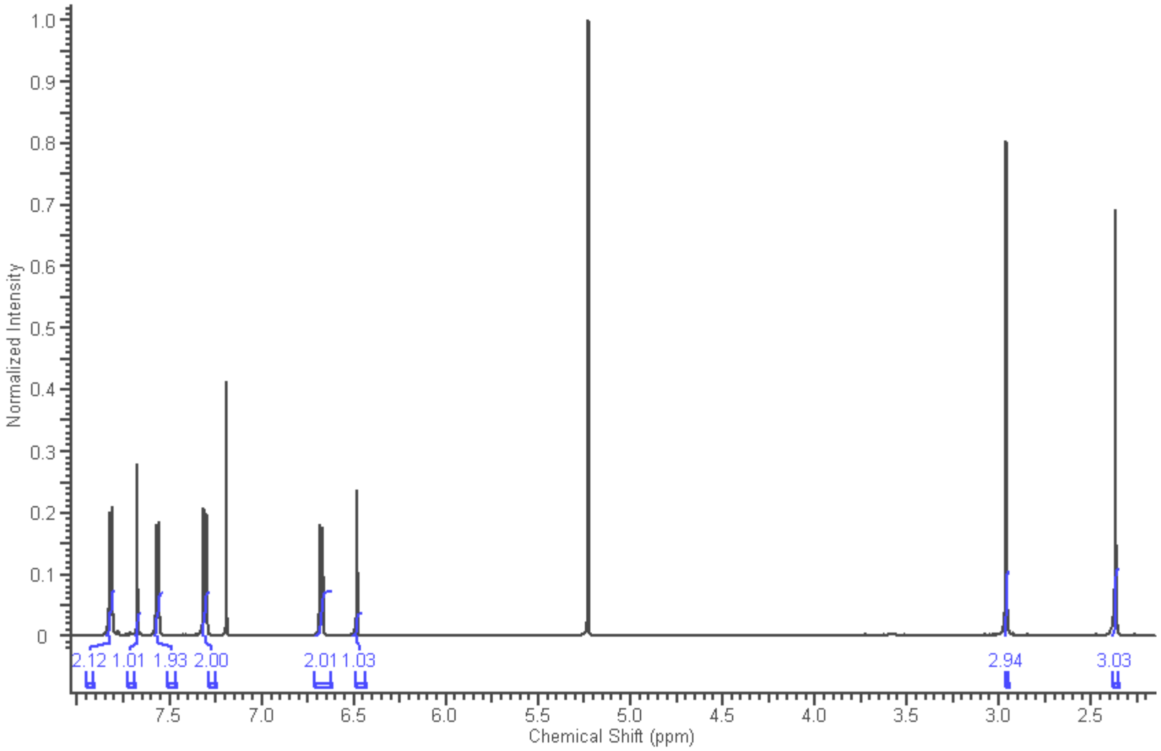

Supplementary Figure 14. ^{1}H NMR spectrum of *in situ* click chemistry hit compound **21**.

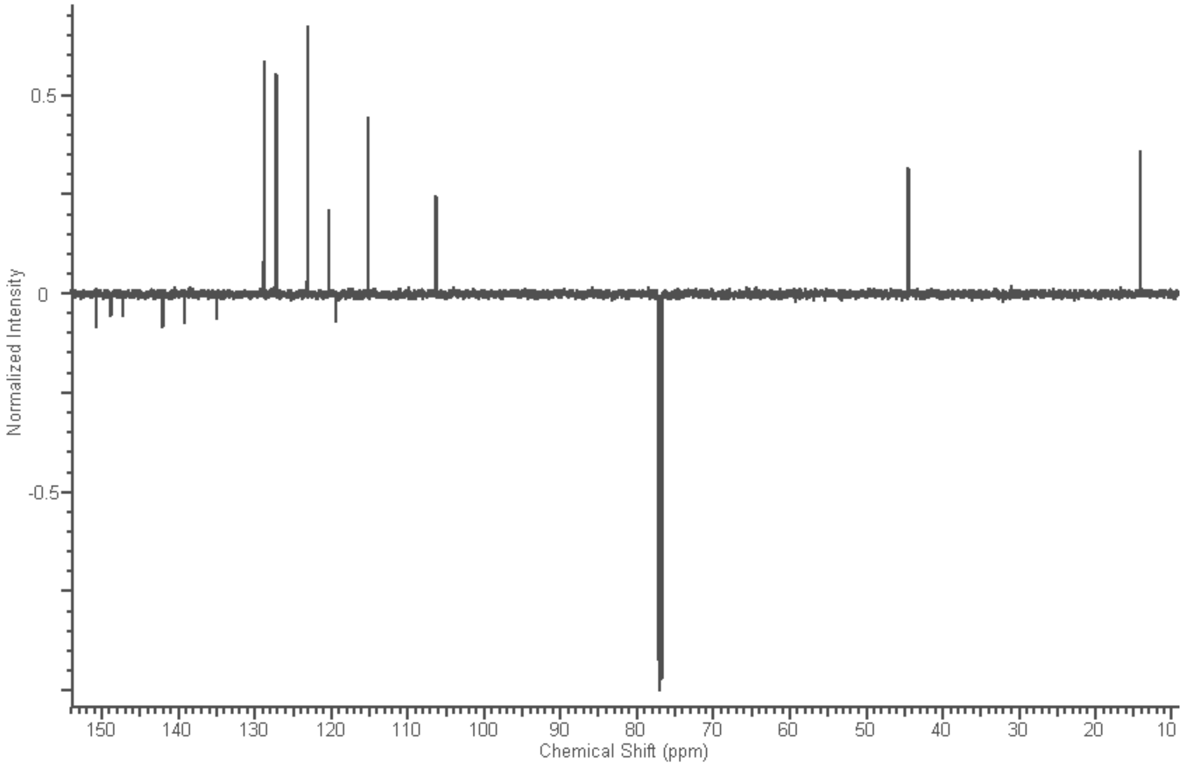

Supplementary Figure 15. ^{13}C NMR spectrum of *in situ* click chemistry hit compound **21**.

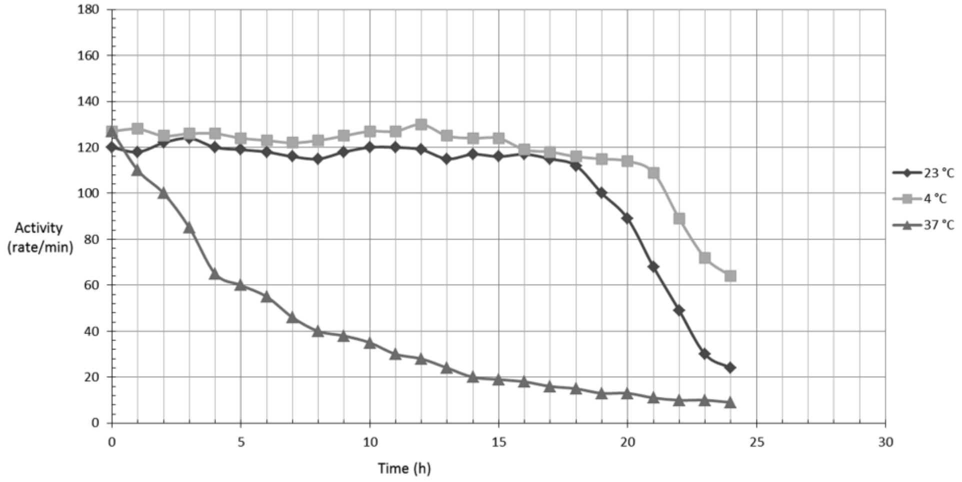

Supplementary Figure 16. COX-2 enzyme activity. Summary of COX-2 activity observed at 4 °C, 27 °C and 37 °C at various time intervals.

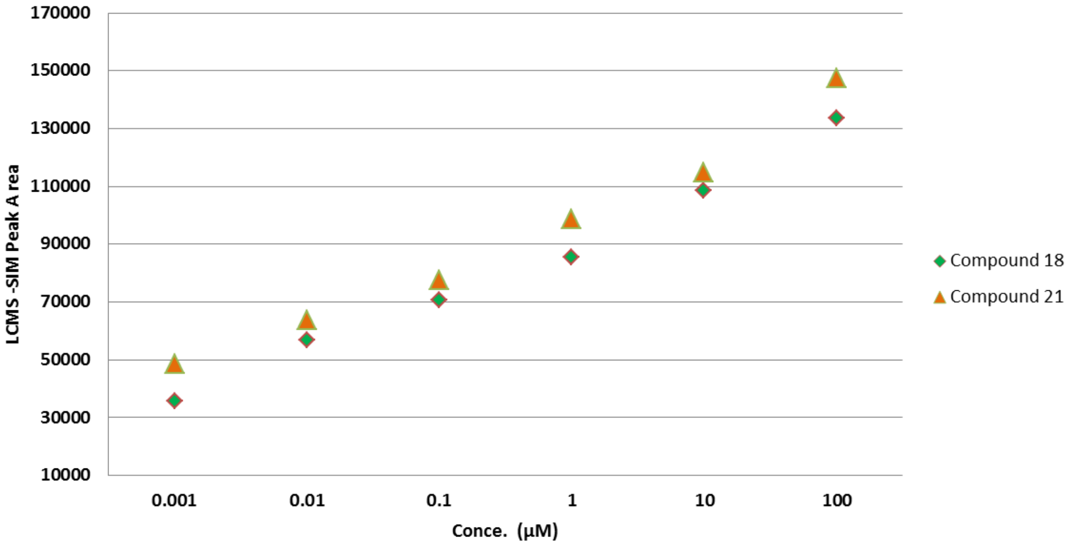

Supplementary Figure 17. Calibration curve for compound 18 and 21. Calibration curve by LC-MS-SIM showing a linear correlation between compound (**18**/**21**) concentration and LC-MS-SIM measured peak area.

Supplementary Table 1. Isothermal titration calorimetry (ITC) data for the titration of azido pyrazole **14** with human COX-2 protein.

**Thermodynamic property**	**Compound 14**
Binding stoichiometry, ***N***	1.02±0.0259
Association constant, $\mathbf{K_a}$	2.20 $x10^{-6}$ M^{-1}
Enthalpy Change, **ΔH**	-34.51 kJ mol^{-1}
Entropy Change at temperature 298.15 K, **TΔS**	1.69 kJ mol^{-1}
Free energy Change, **ΔG**	-36.20 kJ mol^{-1}

Supplementary Table 2. The energy of intermolecular interactions ($E_{intermolecular}$) between ligand and enzyme (COX-2/COX-1) amino acid residues.

Compound	R^1	X	COX-2 $E_{intermolecular}$ (kcal mol^{-1})	COX-1 $E_{intermolecular}$ (kcal mol^{-1})
**7**	H	H	-10.5	-9.7
**8**	Cl	H	-11.5	-7.9
**9**	F	H	-11.7	-8.3
**10**	OCH_3	H	-5.4	-9.4
**11**	CH_3	H	-5.9	-6.7
**12**	CH_2CH_3	H	-4.7	-5.4
**16**	H	SO_2CH_3	-7.2	-6.9
**17**	Cl	SO_2CH_3	-6.9	-5.3
**18**	F	SO_2CH_3	-15.9	-6.9
**19**	OCH_3	SO_2CH_3	-5.9	-7.2
**20**	CH_3	SO_2CH_3	-7.7	-7.4
**21**	NH_2	SO_2CH_3	-16.8	-6.9
**22**	CF_3	SO_2CH_3	-10.9	-7.8
**23**	-	SO_2CH_3	-8.8	-6.5
**24**	-	SO_2CH_3	-11.3	-5.4
**25**	-	SO_2CH_3	-8.5	-5.8
**28**	F	SO_2CH_3	-12.1	-6.1
**29**	NH_2	SO_2CH_3	-7.4	-5.9
**32**	F	H	-5.2	-9.7
**33**	NH_2	H	-6.3	-8.2
**5**	-	SO_2CH_3	-10.1	-7.6
**14**	-	H	-7.5	-7.2
**1**	**Celecoxib**		-14.9	-7.3

Supplementary Table 3. Summary of LC-MS data *in situ* click chemistry screen of 5-azido-1,3-diphenyl-1H-pyrazole (**5**) with alkynes (**6a-6f**, **15a** and **15b**) in presence of COX-2 isozyme.

**Time**	**LC-MS run**	**Alkyne**							
		**6a**	**6b**	**6c**	**6d**	**6e**	**6f**	**15a**	**15b**
**After 6 h**	Run 1 (MS response)	0.2211	0.3455	0.5412	0.8911	0.9854	1.9972	0.9856	1.4225
	Run 2 (MS response)	0.1805	0.4353	0.5232	0.9075	1.1231	1.8902	0.9456	1.5622
	Run 3 (MS response)	0.1989	0.4011	0.5956	0.9301	1.0212	1.6501	0.9012	1.3265
	Average (MS response)	0.2001	0.3939	0.5533	0.9095	1.0428	1.8458	0.9441	1.4370
	Standard Deviation	0.0203	0.0453	0.0376	0.0195	0.0715	0.1777	0.0422	0.1185
**After 9 h**	Run 1 (MS response)	0.2981	0.4569	0.6123	0.9456	1.2654	2.1909	0.8569	1.5369
	Run 2 (MS response)	0.2804	0.5026	0.6345	0.9856	1.2546	2.0311	0.9013	1.4891
	Run 3 (MS response)	0.3199	0.5102	0.6234	0.9021	1.0235	2.1113	0.9845	1.5125
	Average (MS response)	0.2994	0.4899	0.6234	0.9444	1.1811	2.1107	0.9142	1.5128
	Standard Deviation	0.0197	0.0288	0.0111	0.0417	0.1366	0.0804	0.0647	0.0239
**After 12 h**	Run 1 (MS response)	0.2434	0.5963	0.6034	0.8756	0.9953	1.9721	0.8725	1.2354
	Run 2 (MS response)	0.3001	0.6523	0.6462	0.9561	1.0234	2.1936	0.8012	1.1123
	Run 3 (MS response)	0.3142	0.6012	0.5773	0.9654	1.1102	2.211	0.8945	1.3268
	Average (MS response)	0.2859	0.6166	0.6089	0.9323	1.0429	2.1255	0.8560	1.2248
	Standard Deviation	0.0374	0.0310	0.0347	0.0493	0.0598	0.1331	0.0487	0.1076
**After 15 h**	Run 1 (MS response)	0.2701	0.6521	0.4593	0.8963	1.0238	1.9721	0.9156	1.4562
	Run 2 (MS response)	0.2878	0.651	0.5012	0.9129	1.0259	2.1936	0.9812	1.5891
	Run 3 (MS response)	0.3129	0.6021	0.5215	0.9915	1.1263	2.2111	0.9783	1.5689
	Average (MS response)	0.2902	0.6350	0.494	0.9335	1.0586	2.1256	0.9583	1.5380
	Standard Deviation	0.0215	0.0285	0.0258	0.0508	0.0585	0.1332	0.0370	0.0716

Supplementary Table 4. Summary of LC-MS data *in situ* click chemistry screen of 5-azido-1-(4-methanesulfonyl-phenyl)-3-methyl-1H-pyrazole (**14**) with alkynes (**6a-6f** and **15a-15e**) in presence of COX-2 isozyme.

**Time**	**LC-MS run**	**Alkynes**										
		**6a**	**6b**	**6c**	**6d**	**6e**	**6f**	**15a**	**15b**	**15c**	**15d**	**15e**
**After 3 h**	Run 1 (MS response)	0.8592	0.4589	8.2589	0.9978	0.9753	1.5895	8.7956	1.5718	1.2365	1.9456	2.356
	Run 2 (MS response)	0.8503	0.4236	9.5623	0.9123	1.0126	1.5245	9.2356	1.5245	1.2568	1.8956	2.2831
	Run 3 (MS response)	0.8013	0.4125	5.5689	0.9013	1.0356	1.5014	7.8939	1.5123	1.3568	1.856	2.3021
	Average (MS response)	0.8369	0.4316	7.7967	0.9371	1.0076	1.5384	8.6417	1.5362	1.2833	1.8990	2.3137
	Standard Deviations	0.0311	0.0242	2.0364	0.0528	0.0303	0.0456	0.6839	0.0314	0.0644	0.0449	0.0378
**After 6 h**	Run 1 (MS response)	0.9912	0.4789	14.1459	0.9658	1.4587	1.8759	22.1601	1.5012	1.4263	1.995	2.1012
	Run 2 (MS response)	0.9012	0.4893	18.5893	0.9978	1.3012	1.8012	18.165	1.5483	1.4012	1.9025	2.3259
	Run 3 (MS response)	0.9125	0.5123	16.2568	0.9947	1.3201	1.8654	19.1243	1.5478	1.4907	1.9679	2.3925
	Average (MS response)	0.9349	0.4935	16.3306	0.9861	1.3623	1.8475	19.8164	1.5324	1.4394	1.9551	2.2732
	Standard Deviations	0.0490	0.0170	2.2226	0.0176	0.0859	0.0404	2.0855	0.0270	0.0461	0.0388	0.1526
**After 9 h**	Run 1 (MS response)	0.8678	0.5213	49.1989	0.8975	1.3256	1.9014	95.586	1.4796	1.4562	2.0123	1.9923
	Run 2 (MS response)	0.8564	0.4589	46.1569	0.9013	1.0259	1.8956	93.2563	1.4201	1.4027	2.2258	1.9569
	Run 3 (MS response)	0.9012	0.4912	43.1039	0.8942	1.3125	1.9257	91.121	1.4589	1.4014	2.3654	2.0123
	Average (MS response)	0.8751	0.4904	46.1532	0.8976	1.2213	1.9075	93.3211	1.4528	1.4201	2.2011	1.9871
	Standard Deviations	0.0232	0.0312	3.0475	0.0035	0.1693	0.0159	2.2332	0.0302	0.0312	0.1778	0.0280
**After 12 h**	Run 1 (MS response)	0.9562	0.5487	86.1145	0.9785	1.3365	1.9654	93.5126	1.5012	1.5023	2.0459	2.1239
	Run 2 (MS response)	0.9865	0.5125	77.5692	0.9365	1.3518	1.9017	95.2256	1.5246	1.4812	2.1278	2.3212
	Run 3 (MS response)	0.9975	0.5013	78.4562	0.9253	1.3956	1.9987	93.1527	1.5019	1.5823	2.1987	2.2019
	Average (MS response)	0.9800	0.5208	80.7133	0.9467	1.3613	1.9552	93.9636	1.5092	1.5219	2.1241	2.2156
	Standard Deviations	0.0213	0.0247	3.8363	0.0280	0.0306	0.0492	1.10761	0.0133	0.0533	0.0764	0.0993

Supplementary Table 5. Summary of LC-MS data *in situ* click chemistry screen of 5-azido-1,3-diphenyl-1H-pyrazole (**5**), 5-azido-1-(4-methanesulfonyl-phenyl)-3-methyl-1H-pyrazole (**14**), 5-azido-1-(4-methanesulfonyl-phenyl)-3-phenyl-1H-pyrazole (**27**), 5-azido-3-methyl-1-phenyl-1H-pyrazole (**31**) with alkynes (**6a-6f**, **15a** and **15b**) pool in presence of COX-2 isozyme.

Azide	Alkyne	Run 1 (MS response)	Run 2 (MS response)	Run 3 (MS response)	Average (MS response)	Standard Deviation
**5**	**6a**	0.2123	0.2409	0.1759	0.2097	0.0325
**5**	**6b**	0.4895	0.3825	0.4325	0.4348	0.0535
**5**	**6c**	0.4459	0.4058	0.4856	0.4457	0.0399
**5**	**6d**	1.4091	1.5645	1.5025	1.4920	0.0782
**5**	**6e**	2.2561	2.3626	2.3156	2.3114	0.0533
**5**	**6f**	2.4591	2.5125	2.5698	2.5138	0.0553
**5**	**15a**	3.2156	3.1456	3.3256	3.2289	0.0907
**5**	**15b**	0.5256	0.4058	0.5689	0.5001	0.0844
**5**	**15c**	1.3591	1.2314	1.2012	1.2639	0.0838
**5**	**15d**	1.5248	1.4256	1.5941	1.5148	0.0846
**5**	**15e**	2.2048	2.4521	2.3012	2.3193	0.1246
**14**	**6a**	0.3452	0.3526	0.3521	0.3499	0.0041
**14**	**6b**	0.4589	0.4895	0.4587	0.4690	0.0177
**14**	**6c**	88.4891	89.123	91.0914	89.567	1.3569
**14**	**6d**	1.8094	1.8644	1.8045	1.8261	0.0332
**14**	**6e**	2.5401	2.5621	2.4856	2.5292	0.0393
**14**	**6f**	2.9875	2.8789	2.9123	2.9262	0.0556
**14**	**15a**	92.9231	95.9957	97.5893	95.5027	2.3718
**14**	**15b**	1.5323	1.3568	1.5025	1.4638	0.0939
**14**	**15c**	1.5689	1.4789	1.5019	1.5165	0.0467
**14**	**15d**	2.5893	2.5212	2.5437	2.5514	0.0346
**14**	**15e**	2.2758	2.3242	2.3124	2.3041	0.0252
**27**	**6a**	0.2158	0.1859	0.2014	0.2010	0.0149
**27**	**6b**	2.4589	2.5459	2.5651	2.5233	0.0565
**27**	**6c**	2.6598	2.6658	2.6654	2.6636	0.0033
**27**	**6d**	3.5231	3.5562	3.5481	3.5424	0.0172
**27**	**6e**	2.5012	2.5354	2.5689	2.5351	0.0338
**27**	**6f**	2.5481	2.5012	2.5678	2.5390	0.0342
**27**	**15a**	0.2126	0.1889	0.2351	0.2122	0.0231
**27**	**15b**	0.1289	0.1568	0.1645	0.1500	0.0187
**27**	**15c**	4.2123	4.2103	4.2569	4.2265	0.0263
**27**	**15d**	1.3231	1.3321	1.3122	1.3224	0.0099

**27**	**15e**	0.3281	0.3235	0.30125	0.3176	0.0143
**31**	**6a**	1.2349	1.2012	1.1991	1.2117	0.0200
**31**	**6b**	5.2359	5.4523	5.2589	5.3157	0.1188
**31**	**6c**	1.1478	1.2356	1.2012	1.1948	0.0442
**31**	**6d**	3.5012	3.5261	3.5986	3.5419	0.0506
**31**	**6e**	0.3012	0.3331	0.3235	0.3192	0.0163
**31**	**6f**	2.6231	2.6542	2.5891	2.6221	0.0325
**31**	**15a**	1.5123	1.5698	1.5789	1.5536	0.0361
**31**	**15b**	0.4021	0.4356	0.4512	0.4296	0.0250
**31**	**15c**	1.2351	1.2561	1.2012	1.2308	0.0277
**31**	**15d**	2.5012	2.5789	2.5541	2.5447	0.0396
**31**	**15e**	1.8012	1.8562	1.8451	1.8341	0.0290

Supplementary Table 6. Summary of LC-MS data *in situ* click chemistry screen of 5-azido-1,3-diphenyl-1H-pyrazole (**5**) with alkynes (**6a-6f**, **15a** and **15b**) in presence of COX-1 isozyme.

Time	LC-MS Run	Alkynes							
		**6a**	**6b**	**6c**	**6d**	**6e**	**6f**	**15a**	**15b**
**After 6 h**	Run 1 (MS response)	0.3332	1.1231	0.9756	0.8752	0.9756	1.2758	1.3212	1.5426
	Run 2 (MS response)	0.3562	1.1481	0.9012	0.8456	1.0231	1.2364	1.3014	1.5012
	Run 3 (MS response)	0.3254	1.1912	0.9312	0.8012	1.0912	1.3012	1.2656	1.4895
	Average (MS response)	0.3382	1.1541	0.936	0.8406	1.0299	1.2711	1.2960	1.5111
	Standard Deviations	0.0160	0.0344	0.03743	0.0372	0.0581	0.0326	0.0281	0.0279
**After 9 h**	Run 1 (MS response)	0.5856	1.0121	0.8956	0.7896	1.1243	1.2564	2.0123	1.1123
	Run 2 (MS response)	0.5254	1.0197	0.8785	0.7012	1.1945	1.2978	2.0978	1.0756
	Run 3 (MS response)	0.6213	1.123	0.8212	0.7912	1.1023	1.3312	2.0123	1.0326
	Average (MS response)	0.5774	1.0516	0.8651	0.7606	1.1403	1.2951	2.0408	1.0735
	Standard Deviations	0.0484	0.0619	0.0389	0.0515	0.0481	0.0374	0.0493	0.0398
**After 12 h**	Run 1 (MS response)	0.2356	0.8925	0.5642	0.7895	0.7912	0.9783	1.0123	1.4985
	Run 2 (MS response)	0.3021	0.8759	0.6017	0.7045	0.8621	0.9945	1.0856	1.5429
	Run 3 (MS response)	0.3256	0.8045	0.6009	0.8234	0.8517	0.9911	1.0425	1.5913
	Average (MS response)	0.2877	0.8576	0.58893	0.7724	0.8353	0.98796	1.0468	1.5442
	Standard Deviations	0.0466	0.0467	0.0214	0.0612	0.0382	0.0085	0.0368	0.0464
**After 15 h**	Run 1 (MS response)	0.4589	0.7956	0.7589	0.6654	0.9897	1.4512	1.8759	1.023
	Run 2 (MS response)	0.5012	0.7895	0.7986	0.6245	0.9956	1.5014	1.9201	1.1132
	Run 3 (MS response)	0.5124	0.7563	0.7256	0.6012	0.9012	1.5312	1.9013	1.1142
	Average (MS response)	0.4908	0.7804	0.76103	0.6303	0.9621	1.4946	1.8991	1.0834
	Standard Deviations	0.0282	0.0211	0.0298	0.0324	0.0528	0.0404	0.0221	0.0523

Supplementary Table 7. Summary of LC-MS data *in situ* click chemistry screen of 5-azido-1-(4-methanesulfonyl-phenyl)-3-methyl-1H-pyrazole (**14**) with alkynes (**6a-6f** and **15a-15e**) in presence of COX-1 isozyme.

Time	LC-MS Run	Alkynes										
		6a	6b	6c	6d	6e	6f	15a	15b	15c	15d	15e
After 6 h	Run 1 (MS response)	0.3569	1.2451	1.5695	2.0123	1.6581	0.8965	1.2351	1.4225	0.9875	1.3325	2.012
	Run 2 (MS response)	0.3698	1.2458	1.5458	2.1231	1.7895	0.9865	1.2756	1.5622	0.9863	1.2212	2.1121
	Run 3 (MS response)	0.3658	1.2369	1.6012	2.0458	1.7841	0.9458	1.3012	1.3265	0.9952	1.2983	1.9911
	Average (MS response)	0.3641	1.2426	1.5721	2.0604	1.7439	0.9429	1.2553	1.4370	0.9896	1.284	2.0384
	Standard Deviations	0.0066	0.0049	0.0277	0.0568	0.0743	0.0450	0.0286	0.1185	0.0048	0.0570	0.0646
After 9 h	Run 1 (MS response)	0.5879	1.3981	1.2356	2.1236	1.8759	0.7956	1.3201	1.5369	0.9012	1.1253	1.9984
	Run 2 (MS response)	0.5458	1.4012	1.3312	2.1545	1.8012	0.8561	1.3017	1.4891	0.9754	1.8793	1.9975
	Run 3 (MS response)	0.5211	1.3584	1.3013	2.1795	1.8965	0.8756	1.2894	1.5125	0.9215	1.9453	1.9942
	Average (MS response)	0.5516	1.3859	1.2893	2.1525	1.8578	0.8424	1.3037	1.5128	0.9327	1.6499	1.9967
	Standard Deviations	0.0337	0.0238	0.0489	0.0280	0.0501	0.0417	0.0154	0.0239	0.0383	0.3719	0.0022
After 12 h	Run 1 (MS response)	0.8694	1.4251	1.4587	2.0123	1.7589	0.9012	1.2456	1.2354	0.9645	1.3201	1.9856
	Run 2 (MS response)	0.9326	1.4012	1.4012	2.0456	1.7956	0.9758	1.2756	1.1123	0.8893	1.3304	1.9985
	Run 3 (MS response)	0.9012	1.4142	1.3578	2.0356	1.7642	0.9356	1.3014	1.3268	0.9012	1.2893	1.9456
	Average (MS response)	0.9010	1.4135	1.4059	2.0311	1.7729	0.9375	1.2742	1.2248	0.9183	1.3132	1.9765
	Standard Deviations	0.0316	0.0119	0.05061	0.0170	0.0198	0.0373	0.0279	0.1076	0.0404	0.0213	0.0275
After 15 h	Run 1 (MS response)	0.9875	1.3256	1.7583	1.9914	1.8012	0.8365	1.2231	1.4562	1.0125	1.4251	2.1231
	Run 2 (MS response)	0.9012	1.2563	1.8245	2.0127	1.8421	0.8014	1.2014	1.5891	1.0101	1.3856	2.1237
	Run 3 (MS response)	0.9425	1.3216	1.8456	2.0128	1.8301	0.8457	1.2781	1.5689	0.9956	1.3547	2.097
	Average (MS response)	0.9437	1.3011	1.8094	2.0056	1.8244	0.8278	1.2341	1.5380	1.0060	1.3884	2.1146
	Standard Deviations	0.0431	0.0389	0.0371	0.0123	0.0210	0.0233	0.0394	0.0716	0.0091	0.0352	0.0152

Supplementary Methods

***In situ* click chemistry screening in presence of COX-1 isozyme for binary reagent mixtures.** A similar method was employed as described for *in situ* click screening in presence of COX-2 isozyme, except of COX-2 isozyme, COX-1 isozyme (Cayman Chemical Item Number 60100) was used. After 6, 9, 12, 15, 18, 21 and 24 h each sample was analyzed in triplicate by injecting (10 μl) into the LC/MS instrument with selected–ion-monitoring (SIM) mode (Water's Micromass ZQTM 4000 LC-MS instrument, operating in the ESI negative mode, equipped with a Water's 2795 separation module).

Calibration curve for compound 18 and 21. Calibration was performed using authentic compound **18** and **21** under same condition to those used for COX-2 templated *in situ* click chemistry reactions and LCMS-SIM analysis, was also performed in similar fashion (Water's Micromass ZQTM 4000 LCMS instrument, equipped with a Water's 2795 separation module), showed a linear correlation between concentration and response (Supplementary Figure 1). Comparison of the integration values from the *in situ* click chemistry reactions (after 15 h for compound **18** and after 12 h for compound **21** with those from the calibration curve revealed that approximately 0.45 % product **18** and 0.51 % product **21** was formed with respect to the amount of COX-2 enzyme used.

Regioselectivity determination of the *in situ* click chemistry reaction. Upon comparing the retention times of the *in situ* click product (**18**/**21**) with authentic samples, it was revealed that the *in situ* products are *anti*-isomers (Supplementary Figure 2 and 3). Chromatographic and analytical method (LC-MS-SIM) employed for determining the regioisomer distribution was same as it was described monitoring the *in situ* click chemistry reaction.

COX-2 isozyme stability. Both COX isoforms converts arachidonic acid to a prostaglandin G2 (PGG2) intermediate, amount of PGG2 formed is a direct indicator of enzyme activity. The reaction between PGG2 and fluorogenic substrate 10-acetyl-3,7-dihydroxphenoxazine (ADHP) results a fluorescent compound resorufin, amount of resorufin formed can indicate the activity of COX-1/2 enzymes. Here in this experiment, all reagent solutions were prepared by following manufacturer's instructions described in COX fluorescent inhibitor screening assay kit (catalog no. 700100, Cayman Chemical, Ann Arbor, MI). To the 600 μl of assay buffer (100 mM Tris-HCl, pH 8.0) 40 μl of heme and 40 μl COX-2 enzyme was added and samples were stored at 4°C, 23 °C, and 37 °C. At various time intervals, 50 μl of samples were removed and incubated with 10 μl of fluorometric substrate and 10 μl of arachidonic acid (solution prepared by following manufacture's procedure).

Fluorescence emission was measured and relative COX-2 enzyme stability at different temperatures and time intervals was calculated by comparing it with freshly prepared sample. The results, summarized in supplementary figure 4 indicate that COX-2 maintains its activity till 19 h and 21 h at room temperature and 4 °C respectively, however after that COX-2 starts losing its activity. While at 37 °C the COX-2 isozyme was denatured rapidly.

Stability of compound 5, 14, 18 and 21 in human serum and rat serum. Stock solutions of compounds **5**, **14, 18** and **21** (10 mM) were prepared in DMSO for molecular biology. A Solution of each compound (10 mM) was added to freshly thaw human serum (from human male AB plasma, USA origin, sterile-filtered, Sigma–Aldrich) at 37 °C; the final concentration of the compound was 100 µM. The resulting solution was incubated at 37 °C and at various time intervals (0 to 30 h) 200 μL reaction mixture samples was taken and added to 200 µL of acetonitrile containing 0.1% trifluoroacetic acid in order to deproteinize the proteins. Into each sample mixture, 10 μL of an internal standard solution (2 mM solution in methanol) was added. Sample mixture was sonicated, vortexed and then centrifuged for 15 min. at 2150 × g. The clear supernatant was transferred into the fresh tube for LCMS analysis, with a similar method described for the *in situ* click LC-MS studies.

Cellular IC_{50}. Based on the in-vitro COX-2 inhibitory data, compound **5**, **7**, **8**, **9**, **11**, **14**, **17**, **18**, **22**, **24**, **25**, **28**, **29**, **32**, **33** and Celecoxib (**1**) were evaluated of cellular COX-2 inhibitory activity in COX-2 overexpressing live HCA-7 cells (colorectal cancer cell line, COX-2 expression is confirmed with immunoblotting). The HCA-7 cells were cultured in T75 flasks using DMEM/F12 (1:1) medium supplemented with 10% (v/v) fetal bovine serum (GIBCO, 12483), 2 mM L-glutamine (GIBCO, 25030), 1% penicillin/streptomycin and 20 mM HEPES buffer (GIBCO, 15630) and were kept in a 37 ºC humidified incubator with supply of 5% CO2 in air. Cells were washed three times for 5 min with 1 ml PBS. Cells were negatively tested for mycoplasma contamination either by the vendor or in house. The cells were incubated with test compounds, reference drug celecoxib (**1**), or vehicle (solvent) at concentration range of 10^{-9} M to 10^{-3} M. After 30 min incubation the reaction was stopped on ice and supernatants were taken and COX-2 mediated production of PGE2 was immediately determined using prostaglandin E metabolite ELISA kit (514010, Cayman Chemical, Ann Arbor, MI, USA). The PGE2 concentration is quantified by following manufacturer assay procedure. All experiments were done three times and ±s.e.m. were calculated. The known selective COX-2 inhibitor celecoxib was used as a control and for a competition experiment cells were pre-treated with 100 μM celecoxib before the treatment of test compounds. The PRISM5 software was used to calculate IC_{50} values.

Molecular docking procedure. Coordinates from the X-ray crystal structure of COX-1 (ovine, 1EQG, ibuprofen bound in the active site) and COX-2 (murine, 6COX, SC558 bound in the active site) were taken from the RCSB Protein Data Bank server. Compounds were constructed with the builder toolkit of the software package ArgusLab 4.0.1, and energy minimized using the semi-empirical quantum mechanical method PM3. The monomeric structure of the enzyme was chosen, and the active site was defined as 15 Å around the ligand. The molecule to be docked in the enzyme active site was inserted into the workspace carrying the structure of the enzyme. The docking program implements an efficient grid-based docking algorithm, which approximates an exhaustive search within the free volume of the binding site cavity. The conformational space was measured by the geometry optimization of the flexible ligand (rings are treated as rigid) in combination with the incremental construction of the ligand torsions. Thus, docking occurred between the flexible ligand parts of the compound and enzyme. The ligand orientation was determined by a shape scoring function based on A_{score} and the final positions were ranked by lowest interaction energy values. The $E_{interaction}$ value is the sum of the energies involved in hydrogen bond interactions, hydrophobic interactions, and van der Waals interactions. Each molecular docking experiment was repeated three times to confirm the reproducibility. Hydrogen bond and hydrophobic interactions between the compound and enzyme were explored by distance measurements.

Experimental data

Melting points were determined with a Thomas–Hoover capillary apparatus and are uncorrected. ^{1}H NMR (600 MHz) and ^{13}C NMR (150 MHz) spectra were recorded on a Bruker AM-600 NMR spectrometer using $CDCl_3$ or DMSO-d_6 as solvent. Chemical shifts are given in parts per million (ppm) with tetramethylsilane (TMS) as an internal reference and *J* (coupling constant) values were estimated in Hertz (*Hz*). The following notation is used: br – broad, s – singlet, d – doublet, t – triplet, q – quartet, quin – quintet, m – multiplet, dd – doublet of doublets, ddd – doublet of doublets of doublets, dt – doublet of triplets, td – triplet of doublets. Mass spectra (MS) were recorded on a Water's Micromass ZQ 4000 mass spectrometer using the ESI ionization mode. The purity of the compounds was established by elemental analyses, which were performed for C, H, and N by the Microanalytical Service Laboratory, Department of Chemistry, University of Alberta. All target compounds (**7-12** and **16-25**) showed a single spot on Macherey–Nagel Polygram Sil G/UV254 silica gel plates (0.2 mm) using a low, medium, and highly polar solvent system, and no residue remained after combustion, indicating a purity >98%. Column chromatography was performed on a Combiflash Rf system using a gold silica column. All other reagents, purchased from the Aldrich Chemical Co. (Milwaukee, WI, USA), were used without further purification. Compounds **3-5** were synthesized by using previously reported procedures.[1]

General procedure for copper catalyzed alkyne azide cycloaddition (Procedure A). 5-azido-pyrazole (**5/14/27/31**) (0.5 mmol) and alkyne (0.5 mmol) were stirred together in ethanol and water (v/v = 2:1, 5 mL). To this reaction mixture was added copper (II) sulfate dissolved in water (0.5 M, 1 mol %) and aqueous sodium ascorbate (0.1 M, 10 mol %). The reaction mixture was stirred vigorously at room temperature and reaction progress was monitored by TLC. After the completion, the ethanol was removed under reduced pressure and the resulting residue was dissolved ethyl acetate (15 mL) and washed with brine (5 mL). The organic fraction was dried over anhydrous $MgSO_4$ and evaporated under reduced pressure. The crude products were purified by flash column chromatography using a mixture of 2-6 % MeOH in CH_2Cl_2 as eluent to furnish pure title triazole compounds (**7-12**, **16-25**, **28**, **29**, **32** and **33**).

1-(2,5-Diphenyl-2H-pyrazol-3-yl)-4-phenyl-1H-[1,2,3]triazole[1] (**7**). By use of procedure A, the reaction of 5-azido-1,3-diphenyl-1H-pyrazole (**5,** 130 mg, 0.5 mmol) with ethynyl-benzene (**6a,** 0.5 mmol) gave compound **7** in 87% yield as a white solid; mp 122-123 °C (lit.[1] 120 - 121°C). ^{1}H (600 MHz, $CDCl_3$): δ 6.89 (s, 1H, C*H* of pyrazole ring), 7.10-7.25 (m, 6H, Ar-H), 7.27-7.32 (m, 5H, Ar-H), 7.69 (d, *J* = 8.4 Hz, 2H, Ar-H), 7.80 (d, *J* = 8.4 Hz, 2H, Ar-H), 7.92 (s, 1H, C*H* of triazole ring); ^{13}C NMR (150 MHz, $CDCl_3$): δ 103.11 (*C*H of triazole ring), 119.32, 124.64, 126.23, 127.34, 128.54, 129.27, 129.45, 129.89, 129.93, 130.12, 131.21, 132.12, 135.91, 137.93, 147.99, 151.93; ESI-MS: 364.4 $[M+H]^+$; Anal. Calcd for $C_{23}H_{17}N_5$: C, 76.01; H, 4.71; N, 19.27; Found: C, 76.10; H, 4.70; N, 19.23.

4-(4-Chloro-phenyl)-1-(2,5-diphenyl-2H-pyrazol-3-yl)-1H-[1,2,3]triazole (**8**). By use of procedure A, the reaction of 5-azido-1,3-diphenyl-1H-pyrazole (**5,** 130 mg, 0.5 mmol) with 1-chloro-4-ethynyl-benzene (**6b,** 0.5 mmol) gave compound **8** in 88.5% yield as a white solid; mp 134-136 °C; ^{1}H (600 MHz, $CDCl_3$): *δ* 6.38 (s, 1H, C*H* of pyrazole ring), 6.92 (d, *J* = 7.8 Hz, 2H, Ar-H), 7.06-7.29 (m, 6H, Ar-H), 7.35-7.58 (m, 3H, Ar-H), 7.63 (d, *J* = 8.4 Hz, 2H, Ar-H), 7.84 (d, *J* = 8.4 Hz, 2H, Ar-H); ^{13}C NMR (150 MHz, $CDCl_3$): *δ* 104.50 (*C*H of triazole ring), 117.55, 126.16, 126.89, 127.46, 128.13, 128.87, 128.97, 129.71, 129.96, 132.43, 133.29, 134.89, 135.71, 136.92, 147.25, 151.85; ESI-MS: 398.1 $[M(^{35}Cl)+H]^+$; 400.1 $[M(^{37}Cl)+H]^+$; Anal. Calcd for $C_{23}H_{16}ClN_5$: C, 69.43; H, 4.05; N, 17.60; Found: C, 69.38; H, 4.09; N, 17.69.

1-(2,5-Diphenyl-2H-pyrazol-3-yl)-4-(4-fluoro-phenyl)-1H-[1,2,3]triazole (**9**). By use of procedure A, the reaction of 5-azido-1,3-diphenyl-1H-pyrazole (**5,** 130 mg, 0.5 mmol) with 1-ethynyl-4-fluoro-benzene (**6c,** 0.5 mmol) gave compound **9** in 84.5 % yield as a white solid; mp 129-131 °C; ^{1}H (600 MHz, $CDCl_3$): *δ* 6.98 (s, 1H, C*H* of pyrazole ring), 7.03-7.07 (m, 2H, Ar-H), 7.26-7.34 (m, 6H, Ar-H), 7.38-7.41 (m, 2H, Ar-H), 7.69 - 7.72 (m, 2H, Ar-H), 7.85 (d, *J* = 8.4 Hz, 2H, Ar-H), 7.97 (s, 1H, C*H*); ^{13}C NMR (150 MHz, $CDCl_3$): *δ* 102.30 (*C*H of

triazole ring), 116.05 (d, J_{CCF} = 22.5 Hz), 121.46, 123.86, 125.70, 125.80, 127.72, 127.77, 128.65, 128.84 (d, J_{CCCF} = 7.6 Hz), 129.50, 131.99, 135.40, 137.62, 147.22, 151.88, 163.01 (d, J_{CF} = 246 Hz); ESI-MS: 382.4 $[M+H]^+$; Anal. Calcd for $C_{23}H_{16}FN_5$: C, 72.43; H, 4.23; N, 18.36; Found: C, 72.39; H, 4.27; N, 18.39.

1-(2,5-Diphenyl-2H-pyrazol-3-yl)-4-(4-methoxy-phenyl)-1H-[1,2,3]triazole (**10**). By use of procedure A, the reaction of 5-azido-1,3-diphenyl-1H-pyrazole (**5,** 130 mg, 0.5 mmol) with 1-ethynyl-4-methoxy-benzene (**6d**, 0.5 mmol) gave compound **7** in 83% yield as a white solid; mp 117-120 °C; ^{1}H (600 MHz, $CDCl_3$): δ 3.76 (s, 3H, OC*H*$_3$), 6.88 (d, *J* = 8.4 Hz, 2H, Ar-H), 6.96 (s, 1H, C*H*), 7.25-7.30 (m, 4H, Ar-H), 7.32 (d, *J* = 8.1 Hz, 2H, Ar-H), 7.38 (d, *J* = 7.8 Hz, 2H, Ar-H), 7.58 (d, *J* = 8.4 Hz, 2H, Ar-H), 7.84 (d, *J* = 8.4 Hz, 2H, Ar-H), 7.92 (s, 1H, C*H*); ^{13}C NMR (150 MHz, $CDCl_3$): δ 56.60 (O*C*H$_3$), 102.24 (*C*H of triazole ring), 114.40, 120.87, 122.10, 123.81, 125.80, 127.28, 128.57, 128.78, 128.87, 129.46, 132.07, 135.59, 137.68, 147.95, 151.81, 160.07; ESI-MS: 394.4 $[M+H]^+$; Anal. Calcd for $C_{24}H_{19}N_5O$: C, 73.27; H, 4.87; N, 17.80; Found: C, 73.20; H, 4.81; N, 17.84.

1-(2,5-Diphenyl-2H-pyrazol-3-yl)-4-*p*-tolyl-1H-[1,2,3]triazole (**11**). By use of procedure A, the reaction of 5-azido-1,3-diphenyl-1H-pyrazole (**5,** 130 mg, 0.5 mmol) with 1-ethynyl-4-methyl-benzene (**6e**, 0.5 mmol) gave compound **11** in 79.5 % yield as a white solid; mp 125-127 °C; ^{1}H (600 MHz, $CDCl_3$): δ 2.32 (s, 3H, C*H*$_3$), 6.98 (s, 1H, C*H* of pyrazole ring), 7.17 (d, *J* = 7.8 Hz, 2H, Ar-H), 7.26-7.35 (m, 6H, Ar-H), 7.39-7.41 (m, 2H, Ar-H), 7.63 (d, *J* = 8.4 Hz, 2H, Ar-H), 7.85 (d, *J* = 8.4 Hz, 2H, Ar-H), 7.98 (s, 1H, C*H*); ^{13}C NMR (150 MHz, $CDCl_3$): δ 20.73 (*C*H$_3$), 103.50 (*C*H of triazole ring), 117.53, 124.23, 125.86, 125.70, 126.32, 127.13, 128.71, 128.81, 129.65, 129.77, 131.99, 134.10, 135.13, 138.62,147.21, 151.11; ESI-MS: 378.4 $[M+H]^+$; Anal. Calcd for $C_{24}H_{19}N_5$: C, 76.37; H, 5.07; N, 18.55; Found: C, 76.39; H, 5.03; N, 18.58.

1-(2,5-Diphenyl-2H-pyrazol-3-yl)-4-(4-ethyl-phenyl)-1H-[1,2,3]triazole (**12**). By use of procedure A, the reaction of 5-azido-1,3-diphenyl-1H-pyrazole (**5**, 130 mg, 0.5 mmol) with 1-ethyl-4-ethynyl-benzene (**6f**, 0.5 mmol) gave compound **12** in 86.5 % yield as a white solid; mp 130-133 °C; ^{1}H (600 MHz, $CDCl_3$): δ 1.18 (t, *J* =7.8 Hz, 3H, C*H*$_3$), 2.61 (q, *J* = 7.8 Hz, 2H, C*H*$_2$), 6.98 (s, 1H, C*H* of pyrazole ring), 7.19 (d, *J* = 7.8 Hz, 2H, Ar-H), 7.25-7.35 (m, 6H, Ar-H), 7.38-7.41 (m, 2H, Ar-H), 7.65 (d, *J* = 8.4 Hz, 2H, Ar-H), 7.85 (d, *J* = 8.4 Hz, 2H, Ar-H), 7.97 (s, 1H, C*H*); ^{13}C NMR (150 MHz, $CDCl_3$): δ 15.57 (*C*H$_3$), 28.72 (*C*H$_2$), 102.29 (*C*H of triazole ring), 121.39, 123.82, 125.80, 125.96, 126.80, 127.42, 128.60, 128.80, 128.87, 129.51, 132.03, 135.54, 137.61, 145.22, 148.14, 151.83; ESI-MS: 392.4 $[M+H]^+$; Anal. Calcd for $C_{25}H_{21}N_5$: C, 76.70; H, 5.41; N, 17.89; Found: C, 76.72; H, 5.40; N, 17.88.

2-(4-Methanesulfonyl-phenyl)-5-methyl-2H-pyrazol-3-ylamine (**13**). 4-Methanesulfonyl-phenyl)-hydrazine (1 g, 4.50 mmol) was dissolved in ethanol (15 mL) and 3-amino-but-2-enenitrile (0.36 g, 4.50 mmol) was added into the clear solution. The resulting reaction mixture was refluxed for 7 h. The solvent was removed under vacuum; the residue was redissolved in ethyl acetate (200 mL) and washed twice with saturated aqueous $NaHCO_3$ solution (75 ml). The organic layer was dried over anhydrous Na_2SO_4, filtered and solvent was removed under vacuum. The residue was purified by flash chromatography (ethyl acetate : hexane = 7:3) to give pure compound **13** in 95 % yield as light yellow solid; mp 112-114 °C; ^{1}H (600 MHz, CD_3OD): δ 2.19 (s, 3H, CH_3), 3.17 (s, 3H, SO_2CH_3), 5.50 (s, 1H, C*H* of pyrazole ring), 7.86 (d, *J* = 8.4 Hz, 2H, Ar-H), 8.06 (d, *J* = 8.4 Hz, 2H, Ar-H); ^{13}C NMR (150 MHz, CD_3OD): δ 13.61 (*C*H_3), 49.42 (SO_2CH_3), 91.94, 124.36, 128.70, 134.32, 142.82, 149.63, 152.05 (*C*-CH_3); ESI-MS: 252.3 $[M+H]^+$; Anal. Calcd for $C_{11}H_{13}N_3O_2S$: C, 52.57; H, 5.21; N, 16.72; S, 12.76; Found: C, 52.51; H, 5.25; N, 16.69; S, 12.74.

5-Azido-1-(4-methanesulfonyl-phenyl)-3-methyl-1H-pyrazole (**14**)**.** 2-(4-Methanesulfonyl-phenyl)-5-methyl-2H-pyrazol-3-ylamine (**13**, 0.50 g, 2 mmol) was dissolved in 5 mL of trifluoroacetic acid and freshly prepared solution of $NaNO_2$ (0.66 g, 9.5 mmol) in 1 mL of H_2O was added at 0 °C. After stirring for 15 min, a solution of NaN_3 (1.38 g, 21 mmol) in 5 mL of H_2O was added, and the reaction mixture was stirred for 2 h at 20 °C, subjected to extraction with ethyl acetate (3 x 40 mL), washed with H_2O, dried over anhydrous Na_2SO_4, filtered and solvent was removed under vacuum. The residue was purified by flash chromatography (ethyl acetate: hexane = 1:1) to give pure azido compound **14** in 76 % yield as light yellow solid mp 131-133 °C; ^{1}H (600 MHz, CD_3OD): δ 2.24 (s, 3H, CH_3), 3.01 (s, 3H, SO_2CH_3), 6.01 (s, 1H, C*H* of pyrazole ring), 7.69 (d, *J* = 8.4 Hz, 2H, Ar-H), 7.98 (d, *J* = 8.4 Hz, 2H, Ar-H); ^{13}C NMR (150 MHz, CD_3OD): δ 14.17 (*C*H_3), 44.65 (SO_2CH_3), 97.24 (*C*H of pyrazole ring), 123.30, 128.65, 137.83, 138.90, 142.59, 151.03 (*C*-CH_3); ESI-MS: 278.3 $[M+H]^+$; Anal. Calcd for $C_{11}H_{11}N_5O_2S$: C, 47.64; H, 4.00; N, 25.26; S, 11.56; Found: C, 47.60; H, 4.05; N, 25.23; S, 11.52.

1-[2-(4-Methanesulfonyl-phenyl)-5-methyl-2H-pyrazol-3-yl]-4-phenyl-1H-[1,2,3]triazole (**16**)**.** By use of procedure A, the reaction of 5-azido-1-(4-methanesulfonyl-phenyl)-3-methyl-1H-pyrazole (**14**, 138 mg, 0.5 mmol) with ethynyl-benzene (**6a,** 0.5 mmol) gave compound **16** in 86.5 % yield as white solid; mp 146-148 °C; ^{1}H (600 MHz, $CDCl_3$): δ 2.37 (s, 3H, CH_3), 2.95 (s, 3H, SO_2CH_3), 6.51 (s, 1H, C*H* of pyrazole ring), 7.31 (m, 3H, Ar-H), 7.39 (d, *J* = 8.4 Hz, 2H, Ar-H), 7.78 (d, *J* = 8.4 Hz, 2H, Ar-H), 7.83 (d, *J* = 8.4 Hz, 2H, Ar-H), 7.93 (s, 1H, C*H* of triazole ring); ^{13}C NMR (150 MHz, $CDCl_3$): δ 14.07 (*C*H_3), 44.48 (SO_2CH_3), 106.38 (*C*H of pyrazole ring), 123.10, 124.90, 125.97, 128.49, 128.63, 128.74, 129.08, 134.78, 139.35, 141.94, 148.51, 150.84

(C-CH_3); ESI-MS: 380.1 $[M+H]^+$; Anal. Calcd for $C_{19}H_{17}N_5O_2S$: C, 60.14; H, 4.52; N, 18.46; S, 8.45; Found: C, 60.10; H, 4.49; N, 18.49; S, 8.41.

4-(4-Chloro-phenyl)-1-[2-(4-methanesulfonyl-phenyl)-5-methyl-2H-pyrazol-3-yl]-1H-[1,2,3]triazole (**17**). By use of procedure A, the reaction of 5-azido-1-(4-methanesulfonyl-phenyl)-3-methyl-1H-pyrazole (**14**, 138 mg, 0.5 mmol) with 1-chloro-4-ethynyl-benzene (**6b**, 0.5 mmol) gave compound **17** in 90.5 % yield as a white solid; mp 145-148 °C; ^{1}H (600 MHz, $CDCl_3$): δ 2.37 (s, 3H, CH_3), 2.96 (s, 3H, SO_2CH_3), 6.50 (s, 1H, C*H* of pyrazole ring), 7.31 (d, J = 8.4 Hz, 2H, Ar-H), 7.36 (d, J = 8.4 Hz, 2H, Ar-H), 7.71 (d, J = 8.4 Hz, 2H, Ar-H), 7.82 (d, J = 8.4 Hz, 2H, Ar-H), 7.94 (s, 1H, C*H*); ^{13}C NMR (150 MHz, $CDCl_3$): δ 11.72 (*C*H_3), 44.49 (SO_2CH_3), 106.40 (*C*H of pyrazole ring), 121.88, 123.25, 127.21, 127.80, 128.62, 129.53, 134.92, 136.59, 139.41, 141.90, 147.46, 150.87 (C-CH_3); ESI-MS: 414.1 $[M(^{35}Cl)+H]^+$; 416.1 $[M(^{37}Cl)+H]^+$; Anal. Calcd for $C_{19}H_{16}ClN_5O_2S$: C, 55.14; H, 3.90; N, 16.92; S, 7.75; Found: C, 55.17; H, 3.92; N, 16.90; S, 7.71.

4-(4-Fluoro-phenyl)-1-[2-(4-methanesulfonyl-phenyl)-5-methyl-2H-pyrazol-3-yl]-1H-[1,2,3]triazole (**18**). By use of procedure A, the reaction of 5-azido-1-(4-methanesulfonyl-phenyl)-3-methyl-1H-pyrazole (**14**, 138 mg, 0.5 mmol) with 1-ethynyl-4-fluoro-benzene (**6c**, 0.5 mmol) gave **18** in 89.5 % yield as a white solid; mp 129-131 °C; ^{1}H (600 MHz, $CDCl_3$): δ 2.37 (s, 3H, CH_3), 2.96 (s, 3H, SO_2CH_3), 6.51 (s, 1H, C*H* of pyrazole ring), 7.07-7.10 (m, 2H, Ar-H), 7.32 (d, J = 8.4 Hz, 2H, Ar-H), 7.74-7.78 (m, 2H, Ar-H), 7.82 (d, J = 8.4 Hz, 2H, Ar-H), 7.92 (s, 1H, C*H*); ^{13}C NMR (150 MHz, CDC l_3): δ 14.06 (CH_3), 44.49 (SO_2CH_3), 106.38 (*C*H of pyrazole ring), 116.17 (d, J_{CCF} = 21.0 Hz), 121.48, 123.23, 125.30, 127.80 (d, J_{CCCF} = 7.5 Hz), 128.92, 134.70, 139.40, 141.91, 147.64, 150.87 (C-CH_3), 163.12 (d, J_{CF} = 246 Hz); ESI-MS: 398.43 $[M+H]^+$; Anal. Calcd for $C_{19}H_{16}FN_5O_2S$: C, 57.42; H, 4.06; N, 17.62; S, 8.07; Found: C, 57.40; H, 4.03; N, 17.60; S, 8.03.

1-[2-(4-Methanesulfonyl-phenyl)-5-methyl-2H-pyrazol-3-yl]-4-(4-methoxy-phenyl)-1H-[1,2,3]triazole (**19**). By use of procedure A, the reaction of 5-azido-1-(4-methanesulfonyl-phenyl)-3-methyl-1H-pyrazole (**14**, 138 mg, 0.5 mmol) with 1-ethynyl-4-methoxy-benzene (**6d**, 0.5 mmol) gave **19** in 86 % yield as a white solid; mp 124-127 °C; ^{1}H (600 MHz, $CDCl_3$): δ 2.44 (s, 3H, CH_3), 3.00 (s, 3H, SO_2CH_3), 3.86 (s, 3H, OCH_3), 6.57 (s, 1H, C*H* of pyrazole ring), 6.98 (d, J = 7.8 Hz, 2H, Ar-H), 7.39 (d, J = 7.8 Hz, 2H, Ar-H), 7.76 (d, J = 7.8 Hz, 2H, Ar-H), 7.89 (d, J = 7.8 Hz, 2H, Ar-H), 8.01 (s, 1H, C*H*); ^{13}C NMR (150 MHz, $CDCl_3$): δ 14.06 (CH_3), 44.51 (SO_2CH_3), 55.40 (OCH_3), 106.31 (*C*H of pyrazole ring), 114.49, 121.67, 123.18, 127.33, 128.49, 128.91, 134.88, 139.29, 141.96, 148.38, 150.82 (C-CH_3) 160.26 (Ar*C*-OCH_3); ESI-MS: 394.1 $[M+H]^+$; Anal. Calcd for $C_{20}H_{19}N_5O_2S$: C, 61.05; H, 4.87; N, 17.80; S, 8.15; Found: C, 61.09; H, 4.81; N, 17.89; S, 8.10.

1-[2-(4-Methanesulfonyl-phenyl)-5-methyl-2H-pyrazol-3-yl]-4-p-tolyl-1H-[1,2,3]triazole (**20**). By use of procedure A, the reaction of 5-azido-1-(4-methanesulfonyl-phenyl)-3-methyl-1H-pyrazole (**14**, 138 mg, 0.5 mmol) with 1-ethynyl-4-methyl-benzene (**6e**, 0.5 mmol) gave compound **16** in 82 % yield as a white solid; mp 144-146 °C; ^{1}H (600 MHz, $CDCl_3$): δ 2.32 (s, 3H, CH_3), 2.37 (s, 3H, CH_3), 2.96 (s, 3H, SO_2CH_3), 6.50 (s, 1H, C*H* of pyrazole ring), 7.19 (d, *J* = 7.8 Hz, 2H, Ar-H), 7.31 (d, *J* = 8.4 Hz, 2H, Ar-H), 7.65 (d, *J* = 7.8 Hz, 2H, Ar-H), 7.82 (d, *J* = 8.4 Hz, 2H, Ar-H), 7.98 (s, 1H, C*H*); ^{13}C NMR (150 MHz, $CDCl_3$): δ 11.91 (*C*H_3), 21.29 (CH_3), 44.51 (SO_2CH_3), 106.34 (*C*H of pyrazole ring), 121.43, 123.18, 125.86, 126.24, 128.64, 129.76, 134.85, 139.11, 139.30, 141.96, 148.59, 150.82 (*C*-CH_3); ESI-MS: 394.1 $[M+H]^+$; Anal. Calcd for $C_{20}H_{19}N_5O_2S$: C, 61.05; H, 4.87; N, 17.80; S, 8.15; Found: C, 61.09; H, 4.81; N, 17.89; S, 8.10.

4-{1-[2-(4-Methanesulfonyl-phenyl)-5-methyl-2H-pyrazol-3-yl]-1H-[1,2,3]triazol-4-yl}-phenylamine (**21**). By use of procedure A, the reaction of 5-azido-1-(4-methanesulfonyl-phenyl)-3-methyl-1H-pyrazole (**14**, 138 mg, 0.5 mmol) with 4-ethynyl-phenylamine (**15a**, 0.5 mmol) gave compound **21** in 87.5 % yield as a white solid; mp 177-180 °C; ^{1}H (600 MHz, $CDCl_3$): δ 2.36 (s, 3H, CH_3), 3.00 (s, 3H, SO_2CH_3), 6.48 (s, 1H, C*H* of pyrazole ring), 6.65 (d, *J* = 8.4 Hz, 2H, Ar-H), 7.31 (d, *J* = 8.4 Hz, 2H, Ar-H), 7.83 (d, *J* = 8.4 Hz, 2H, Ar-H), 7.84 (d, *J* = 8.4 Hz, 2H, Ar-H), 8.10 (s, 1H, C*H*); ^{13}C NMR (150 MHz, $CDCl_3$): δ 14.07 (*C*H_3), 42.52 (SO_2CH_3), 106.26 (*C*H of pyrazole ring), 115.25, 119.36, 121.52, 123.49, 128.22, 132.41, 135.36, 139.73, 141.94, 147.25, 148.87, 150.78 (*C*-CH_3); ESI-MS: 395.1 $[M+H]^+$; Anal. Calcd for $C_{19}H_{18}N_6O_2S$: C, 57.85; H, 4.60; N, 21.31; S, 8.13; Found: C, 57.82; H, 4.65; N, 21.34; S, 8.10.

1-[2-(4-Methanesulfonyl-phenyl)-5-methyl-2H-pyrazol-3-yl]-4-(4-trifluoromethyl-phenyl)-1H-[1,2,3]triazole (**22**). By use of procedure A, the reaction of 5-azido-1-(4-methanesulfonyl-phenyl)-3-methyl-1H-pyrazole (**14**, 138 mg, 0.5 mmol) with 1-ethynyl-4-trifluoromethyl-benzene (**15b**, 0.5 mmol) gave compound **22** in 87.5 % yield as a light yellow solid; mp 209-211 °C; ^{1}H (600 MHz, CD_3OD): δ 2.46 (s, 3H, *C*H_3), 3.14 (s, 3H, SO_2CH_3), 6.81 (s, 1H, C*H* of pyrazole ring), 7.14-7.38 (m, 2H, Ar-H), 7.46-7.48 (m, 2H, Ar-H), 7.78-7.88 (m, 2H, Ar-H), 7.98-8.11 (m, 2H, Ar-H), 8.14 (s, 1H, C*H*); ^{13}C NMR (150 MHz, CD_3OD): δ 12.39 (*C*H_3), 42.74 (SO_2CH_3), 105.57 (*C*H of pyrazole ring), 119.12 (q, $^1J_{C,F}$ = 269 Hz, *C*F_3), 123.57, 124.50, 125.62, 127.06 128.92, 136.9 (q, $^2J_{CCF}$ = 15 Hz, *C*-CF_3), 137.5, 140.25, 141.09, 146.52, 150.89, 151.87 (*C*-CH_3); ESI-MS: 398.43 $[M+H]^+$; Anal. Calcd for $C_{20}H_{16}F_3N_5O_2S$: C, 53.69; H, 3.60; N, 15.65; S, 7.17; Found: C, 53.64; H, 3.64; N, 15.60; S, 7.19.

4-Isobutyl-1-[2-(4-methanesulfonyl-phenyl)-5-methyl-2H-pyrazol-3-yl]-1H-[1,2,3]triazole (**23**). By use of procedure A, the reaction of 5-azido-1-(4-methanesulfonyl-phenyl)-3-methyl-1H-pyrazole (**14**, 138 mg, 0.5

mmol) with 4-methylpent-1-yne (**15c**, 0.5 mmol) gave compound **23** in 79 % yield as a white solid; mp 150-153 °C; ^{1}H (600 MHz, CD_3OD): δ 0.94 (d, J = 6.6 Hz, 6H, 2xCH_3), 1.97 (m, 1H, CH), 2.43 (s, 3H, CH_3), 2.63 (d, J = 6.6 Hz, 2H, CH_2), 3.13 (s, 3H, CH_3), 6.74 (s, 1H, CH of pyrazole ring), 7.34-7.37 (m, 2H, Ar-H), 7.95-7.97 (m, 2H, Ar-H), 8.03 (s, 1H, CH); ^{13}C NMR (150 MHz, CD_3OD): δ 13.87 (CH$_3$), 22.35 (CH$_3$), 22.46 (CH$_3$), 29.81 (CH), 35.04 (CH$_2$), 44.21 (SO_2CH_3), 106.85 (CH of pyrazole ring), 124.82, 126.62, 129.82, 136.83, 141.30, 143.20, 148.92, 152.22 (C-CH$_3$); ESI-MS: 360.1 $[M+H]^+$; Anal. Calcd for $C_{17}H_{21}N_5O_2S$: C, 56.80; H, 5.89; N, 19.48; S, 8.92; Found: C, 56.85; H, 5.84; N, 19.45; S, 8.96.

4-{1-[2-(4-Methanesulfonyl-phenyl)-5-methyl-2H-pyrazol-3-yl]-1H-[1,2,3]triazol-4-ylmethyl}-morpholine (**24**). By use of procedure A, the reaction of 5-azido-1-(4-methanesulfonyl-phenyl)-3-methyl-1H-pyrazole (**14**, 0.13 g, 0.5 mmol) with 4-(prop-2-ynyl)morpholine (**15d**, 0.5 mmol) gave compound **24** in 78.5 % yield as a white solid; mp 172-174 °C; ^{1}H (600 MHz, $CDCl_3$): δ 2.43 (s, 3H, CH$_3$), 2.51-2.76 (m, 4H, 2xCH$_2$), 3.06 (s, 3H, CH$_3$), 3.47-3.50 (m, 6H, 3xCH$_2$), 6.58 (s, 1H, CH of pyrazole ring), 7.27 (d, J = 8.4, 2H, Ar-H), 7.89 (d, J = 8.4, 2H, Ar-H), 8.11 (s, 1H, CH); ^{13}C NMR (150 MHz, $CDCl_3$): δ 14.04 (CH$_3$), 44.49 (SO$_2$$CH_3$), 53.06 ($CH_2$), 64.06 ($CH_2$), 75.82 ($CH_2$), 106.36 ($C$H of pyrazole ring), 123.34, 126.72, 129.00, 134.73, 139.46, 141.82, 148.72, 150.85 (C-CH$_3$); ESI-MS: 403.1 $[M+H]^+$; Anal. Calcd for $C_{18}H_{22}N_6O_3S$: C, 53.72; H, 5.51; N, 20.88; S, 7.97, Found: C, 53.78; H, 5.48; N, 20.82; S, 7.94.

1-{1-[2-(4-Methanesulfonyl-phenyl)-5-methyl-2H-pyrazol-3-yl]-1H-[1,2,3]triazol-4-ylmethyl}-piperidine (25). By use of procedure A, the reaction of 5-azido-1-(4-methanesulfonyl-phenyl)-3-methyl-1H-pyrazole (**14**, 138 mg, 0.5 mmol) with 1-prop-2-ynyl-piperidine (**15e**, 0.5 mmol) gave compound **25** in 80 % yield as a colorless thick liquid; ^{1}H (600 MHz, $CDCl_3$): δ 1.57-1.59 (m, 2H, CH_2), 1.77-1.79 (m, 4H, 2xCH_2), 2.43 (s, 3H, CH_3), 2.84-2.88 (m, 4H, 2xCH$_2$), 3.06 (s, 3H, CH_3), 4.06 (s, 2H, CH_2), 6.57 (s, 1H, CH of pyrazole ring), 7.31 (d, J = 8.4, 2H, Ar-H), 7.88 (d, J = 8.4, 2H, Ar-H), 8.00 (s, 1H, CH); ^{13}C NMR (150 MHz, CD_3OD): δ 14.13 (CH$_3$), 22.76 (CH$_2$), 23.88 (CH$_2$), 44.50 (SO$_2$$CH_3$), 52.25 (CH$_2$), 53.56 ($CH_2$), 106.38 ($C$H of pyrazole ring), 123.68, 127.92, 128.75, 129.17, 134.44, 139.48, 141.77, 150.88 (C-CH$_3$); ESI-MS: 401.1 $[M+H]^+$; Anal. Calcd for $C_{19}H_{24}N_6O_2S$: C, 56.98; H, 6.04; N, 20.98; S, 8.01, Found: C, 56.94; H, 6.08; N, 20.93; S, 8.06.

2-(4-Methanesulfonyl-phenyl)-5-phenyl-2H-pyrazol-3-ylamine (**26**). 1-Amino-1-phenylacrylonitrile[1] (**3**) (0.72 g, 5 mmol) was suspended in 2.5 N HCl (10 mL, 5 mmol) and heated to 50 °C for 5 min. [4-(Methylsulfonyl)phenyl]hydrazine hydrochloride (1.3 g, 6 mmol) was added, followed by 12 N HCl (2.5 mL, 30 mmol), and the mixture was heated to 110 °C for 20 min. The reaction mixture was cooled and 14 N NH_4OH was added dropwise until the solution became basic (7 mL, 100 mmol). Extracted with ethyl acetate (3x100

mL), washed with water and organic layer were combined and dried over (Na_2SO_4), the solvent was evaporated to give crude. Recrystallization from *i*-Pr_2O (15 mL) gave compound **26** in 49 % yield as light yellow solid; mp 113-115 °C; 1H (600 MHz, DMSO-d_6): δ 2.86 (s, 3H, SO_2CH_3), 5.90 (s, 1H, C*H* of pyrazole ring), 7.28-7.39 (m, 3H, Ar-H), 7.42-7.47 (m, 2H, Ar-H), 7.50 (d, *J* = 7.8, 2H, Ar-H), 7.92 (d, *J* = 7.8, 2H, Ar-H); ^{13}C NMR (150 MHz, DMSO-d_6): δ 46.42 (SO_2CH_3), 90.52 (*C*H of pyrazole ring), 124.41, 127.53, 128.65, 129.27, 130.18, 134.32, 138.64, 146.42, 149.25, 152.05 (*C*-CH_3); ESI-MS: 314.1 $[M+H]^+$.

5-Azido-1-(4-methanesulfonyl-phenyl)-3-phenyl-1H-pyrazole (**27**). 2-(4-Methanesulfonyl-phenyl)-5-phenyl-2H-pyrazol-3-ylamine (**26**, 1.31 g, 4.2 mmol) was dissolved in 5 mL of trifluoroacetic acid and a solution of $NaNO_2$ (1.31 g, 19 mmol) in 2 mL of H_2O was added at 0 °C. After stirring the reaction mixture for 15 min, a solution of NaN_3 (2.76 g, 42 mmol) in 8 mL of H_2O was added, and the mixture was stirred for 40 min at 20 °C. The reaction mixture was extracted with ethyl acetate (3x30 mL), washed with H_2O and brine, dried over Na_2SO4. The combined organic layers were evaporated and purified by flash chromatography in hexanes-ethyl acetate (7:3) to give title compound in 76 % yield as light yellow solid; mp 132-134 °C; ^1H (600 MHz, DMSO-d_6): δ 2.90 (s, 3H, CH_3), 6.10 (s, 1H, C*H* of pyrazole ring), 7.23-7.33 (m, 3H, Ar-H), 7.45-7.49 (m, 2H, Ar-H), 7.50 (d, *J* = 7.8, 2H, Ar-H), 7.90 (d, *J* = 7.8, 2H, Ar-H); ^{13}C NMR (150 MHz, DMSO-d6): δ 46.42 (SO_2CH_3), 105.21(*C*H of pyrazole ring), 123.31, 126.42, 129.61, 130.23, 130.81, 134.59, 138.89, 146.97, 149.21, 151.23 (*C*-CH_3); ESI-MS: 340.1 $[M+H]^+$.

4-(4-Fluoro-phenyl)-1-[2-(4-methanesulfonyl-phenyl)-5-phenyl-2H-pyrazol-3-yl]-1H-[1,2,3]triazole (**28**). By use of procedure A, the reaction of 5-azido-1-(4-methanesulfonyl-phenyl)-3-phenyl-1H-pyrazole (**27**, 0.19 g, 0.5 mmol) with 1-ethynyl-4-fluoro-benzene (**6c,** 0.5 mmol) gave compound **28** in 79 % yield as a colorless thick liquid; ^1H (600 MHz, $CDCl_3$): δ 2.93 (s, 3H, SO_2CH_3), 6.43 (s, 1H, C*H* of pyrazole ring), 7.07-7.10 (m, 2H, Ar-H), 7.26-7.36 (m, 3H, Ar-H), 7.40-7.45 (m, 2H, Ar-H), 7.49-7.57 (m, 4H, Ar-H), 7.86-7.90 (m, 2H, Ar-H), 8.04 (s, 1H, C*H* of triazole ring); ^{13}C NMR (150 MHz, $CDCl_3$): δ 46.64 (SO_2CH_3), 106.04 (*C*H of pyrazole ring), 116.28 (d, J_{CCF} = 21.0 Hz), 120.31, 125.36 126.72, 126.99 (d, J_{CCCF} = 7.5 Hz), 128.34, 128.85, 129.56, 130.11, 131.01, 134.56, 136.59, 139.34, 146.89, 150.87 (*C*-CH_3), 163.14 (d, J_{CF} = 246 Hz); ESI-MS: 460.1$[M+H]^+$. Anal. Calcd for $C_{24}H_{18}FN_5O_2S$: C, 62.73; H, 3.95; N, 15.24; S, 6.98; Found: C, 62.70; H, 3.93; N, 15.28; S, 6.91.

4-{1-[2-(4-Methanesulfonyl-phenyl)-5-phenyl-2H-pyrazol-3-yl]-1H-[1,2,3]triazol-4-yl}-phenylamine (**29**). By use of procedure A, the reaction of 5-azido-1-(4-methanesulfonyl-phenyl)-3-phenyl-1H-pyrazole (**27,** 0.19 g, 0.5 mmol) with 4-ethynyl-phenylamine (**15a**) gave compound **29** in 81 % yield as a thick yellow liquid; ^1H

(600 MHz, $CDCl_3$): δ 2.95 (s, 3H, CH_3), 6.49 (s, 1H, C*H* of pyrazole ring), 6.79 (d, J = 8.4 Hz, 2H, Ar-H), 7.23 (d, J = 8.4 Hz, 2H, Ar-H), 7.27-7.34 (m, 3H, Ar-H), 7.43-7.49 (m, 2H, Ar-H), 7.51-7.58 (m, 2H, Ar-H), 7.82-7,89 (m, 2H, Ar-H), 8.05 (s, 1H, C*H*); ^{13}C NMR (150 MHz, $CDCl_3$): δ 46.24 (SO_2CH_3), 105.85 (*C*H of pyrazole ring), 115.60, 119.31, 123.36 126.72, 127.04, 127.89, 128.85, 129.56, 130.11, 131.01, 134.56, 136.59, 139.34, 146.89, 148.72, 150.87 (*C*-CH_3); ESI-MS: 457.1 $[M+H]^+$. Anal. Calcd for $C_{24}H_{20}N_6O_2S$: C, 63.14; H, 4.42; N, 18.41; S, 7.02; Found: C, 63.10; H, 4.47; N, 18.47; S, 7.08.

5-Methyl-2-phenyl-2H-pyrazol-3-ylamine[2] (**30**). By following same procedure as described for the synthesis of compound **26**, the reaction of 1-amino-1-phenylacrylonitrile (**3**) with phenyhydrazine hydrochloride gave compound **30** in 52 % yield as white solid, mp 113-114 °C (lit.[2] mp : 111-112 °C); ^1H (600 MHz, $CDCl_3$): δ 2.28 (s, 3H, CH_3), 5.78 (s, 1H, C*H* of pyrazole ring), 7.30-7.40 (m, 3H, Ar-H), 7.56 (d, J = 7.8, 2H, Ar-H); ESI-MS: 174.1 $[M+H]^+$.

5-Azido-3-methyl-1-phenyl-1H-pyrazole (**31**). By following same procedure as described for the synthesis of compound **27**, the reaction of 5-methyl-2-phenyl-2H-pyrazol-3-ylamine (**30**) with $NaNO_2$ and NaN_3 gave compound **31** in 56 % yield as white solid, mp 123-125 °C. ^1H (600 MHz, $CDCl_3$): δ 2.41 (s, 3H, CH_3), 6.09 (s, 1H, C*H* of pyrazole ring), 7.28-7.43 (m, 3H, Ar-H), 7.59 (d, J = 7.8, 2H, Ar-H). ESI-MS: 200.1 $[M+H]^+$.

4-(4-Fluoro-phenyl)-1-(5-methyl-2-phenyl-2H-pyrazol-3-yl)-1H-[1,2,3]triazole (**32**). By use of procedure A, the reaction of 5-azido-3-methyl-1-phenyl-1H-pyrazole (**31**, 0.09 g, 0.5 mmol) with 1-ethynyl-4-fluoro-benzene (**6c**, 0.5 mmol) gave compound **32** in 81 % yield as colorless thick liquid; (600 MHz, $CDCl_3$): δ 2.49 (s, 3H, CH_3), 6.68 (s, 1H, C*H* of pyrazole ring), 6.96 (d, J = 8.4 Hz, 2H, Ar-H), 7.32-7.36 (m, 3H, Ar-H), 7.39-7.44 (m, 2H, Ar-H), 7.48 (d, J = 8.4 Hz, 2H, Ar-H), 7.94 (s, 1H, C*H*); ^{13}C NMR (150 MHz, $CDCl_3$): δ 13.41 (*C*H_3), 107.01 (*C*H of pyrazole ring), 116.21 (d, J_{CCF} = 21.0 Hz), 119.38, 123.36 126.72, 127.75 (d, J_{CCCF} = 7.5 Hz), 127.99, 128.85, 129.86, 134.97, 137.34, 150.77 (*C*-CH_3), 163.19 (d, J_{CF} = 246.1 Hz); ESI-MS: 320.1 $[M+H]^+$. Anal. Calcd for $C_{18}H_{14}FN_5$: C, 67.70; H, 4.42; N, 21.93; Found: C, 67.64; H, 4.47; N, 21.88.

4-[1-(5-Methyl-2-phenyl-2H-pyrazol-3-yl)-1H-[1,2,3]triazol-4-yl]-phenylamine (**33**). By use of procedure A, the reaction of 5-azido-3-methyl-1-phenyl-1H-pyrazole (**31**, 0.09 g, 0.5 mmol) with 4-ethynyl-phenylamine (**15a**, 0.5 mmol) gave compound **33** in 82 % yield as colorless thick liquid; ^1H (600 MHz, $CDCl_3$): δ 2.43 (s, 3H, CH_3), 6.61 (s, 1H, C*H* of pyrazole ring), 6.84 (d, J = 8.4 Hz, 2H, Ar-H), 7.21 (d, J = 8.4 Hz, 2H, Ar-H), 7.29-7.34 (m, 3H, Ar-H), 7.37-7.42 (m, 2H, Ar-H), 7.95 (s, 1H, C*H*); ^{13}C NMR (150 MHz, $CDCl_3$): δ 13.01 (*C*H_3), 107.01 (*C*H of pyrazole ring), 115.81, 118.88, 124.26 126.72, 127.05, 128.14, 128.11, 129.99, 131.01,

137.34, 147.2, 150.19 (*C*-CH_3); ESI-MS: 317.1 $[M+H]^+$. Anal. Calcd for $C_{18}H_{16}N_6$: C, 68.34; H, 5.10; N, 26.56; Found: C, 68.30; H, 5.12; N, 26.58.

Supplementary References

1 de Paulis, T. *et al*. Substituent effects of *N*-(1,3-diphenyl-1*H*-pyrazol-5-yl)benzamides on positive allosteric modulation of the metabotropic glutamate-5 receptor in rat cortical astrocytes. *J. Med. Chem.* **49**, 3332-3344, (2006).

2 Ganesan, A. & Heathcock, C. H. Synthesis of unsymmetrical pyrazines by reaction of an oxadiazinone with enamines. *J. Org. Chem.* **58**, 6155-6157, (1993).
